# Supplementary material for: Imputing single-cell protein abundance in multiplex tissue imaging
Source: Nat Commun. 2025 May 22;16:4747. doi: 10.1038/s41467-025-59788-x (PMC12098973; doi:10.1038/s41467-025-59788-x)
Supplement: Supplementary file 1 — Supplementary Information [file 41467_2025_59788_MOESM1_ESM.pdf]

Supplementary Information

Network	0 µm/ Baseline	15 µm	30 µm	60 µm	90 µm	120 µm
LGBM	0.099 (0.060)	0.101 (0.059)	0.101 (0.059)	0.097 (0.055)	0.104 (0.062)	0.106 (0.068)
AE Single Protein	0.128 (0.089)	0.100 (0.064)	0.105 (0.059)	0.113 (0.062)	0.120 (0.076)	0.119 (0.075)
AE Multi Protein	0.120 (0.087)	0.115 (0.068)	0.112 (0.062)	0.117 (0.068)	0.120 (0.081)	0.121 (0.081)

Supplementary Table 1: Mean and (Standard Deviation) of all observed radii as well as the baseline for LGBM, AE Single and Multi-Imputation using an Across-Patient Setup. Source data are provided as a Source Data file.

Protein	Variance
CK19	1.28
Vimentin	0.42
ER	0.32
pERK	0.24
aSMA	0.22
pRB	0.18
Ecad	0.14
AR	0.13
CK14	0.11
CD45	0.09
HER2	0.09
Ki67	0.08
EGFR	0.06
P21	0.05
PR	0.05
CK17	0.05

Supplementary Table 2: Variance for each observed protein: CK19 shows to highest variance across all patients, followed by Vimentin and ER. Lowest Variance can be observed for Proteins p21,PR and CK17. Source data are provided as a Source Data file.

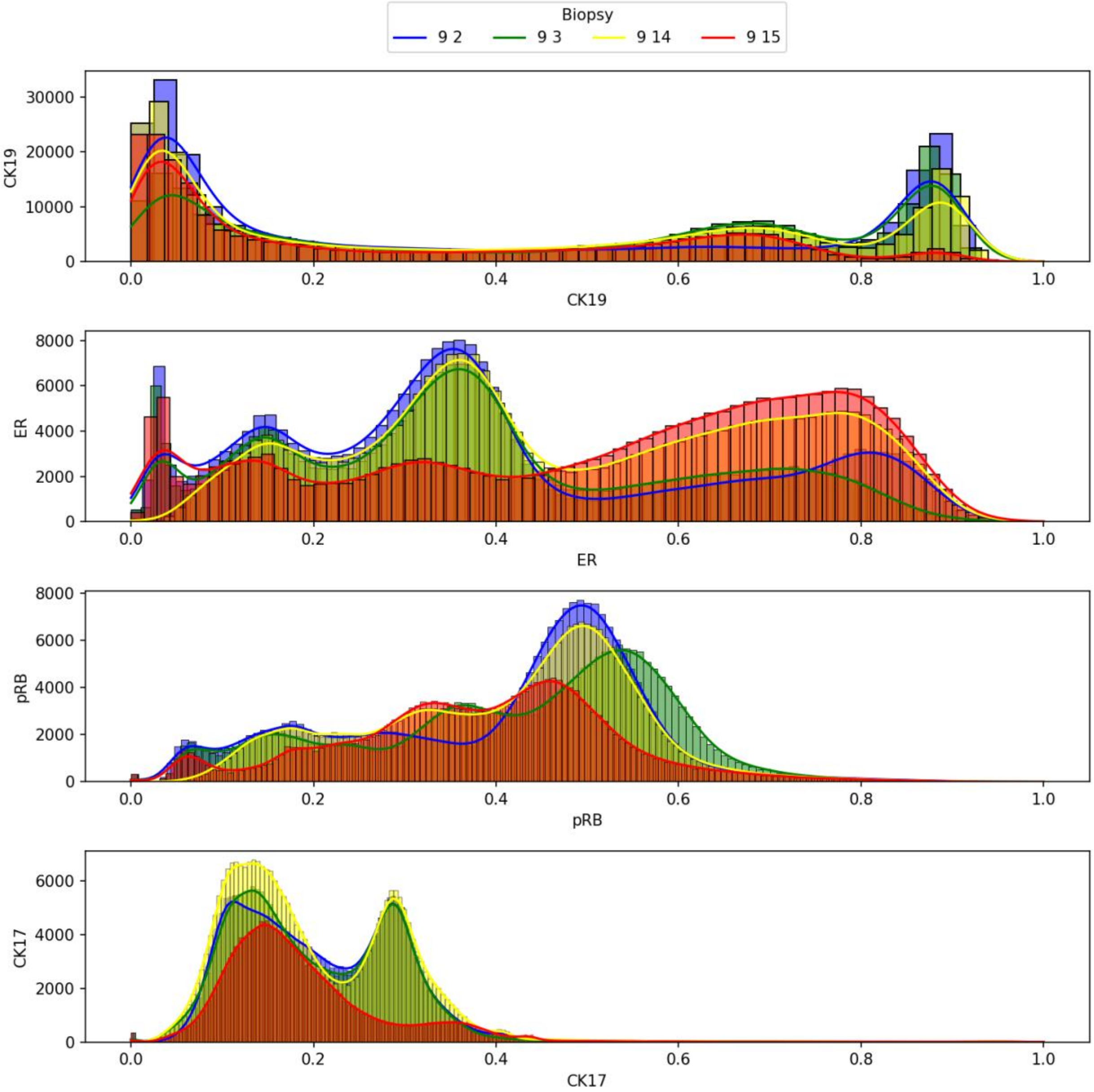

Supplementary Figure 1: Protein expression distribution for four proteins (CK19, ER, pRB, CK17). Proteins CK19 and ER experience the highest variance. The distribution is heterogeneous across both patients and proteins. Source data is provided as a Source Data file.

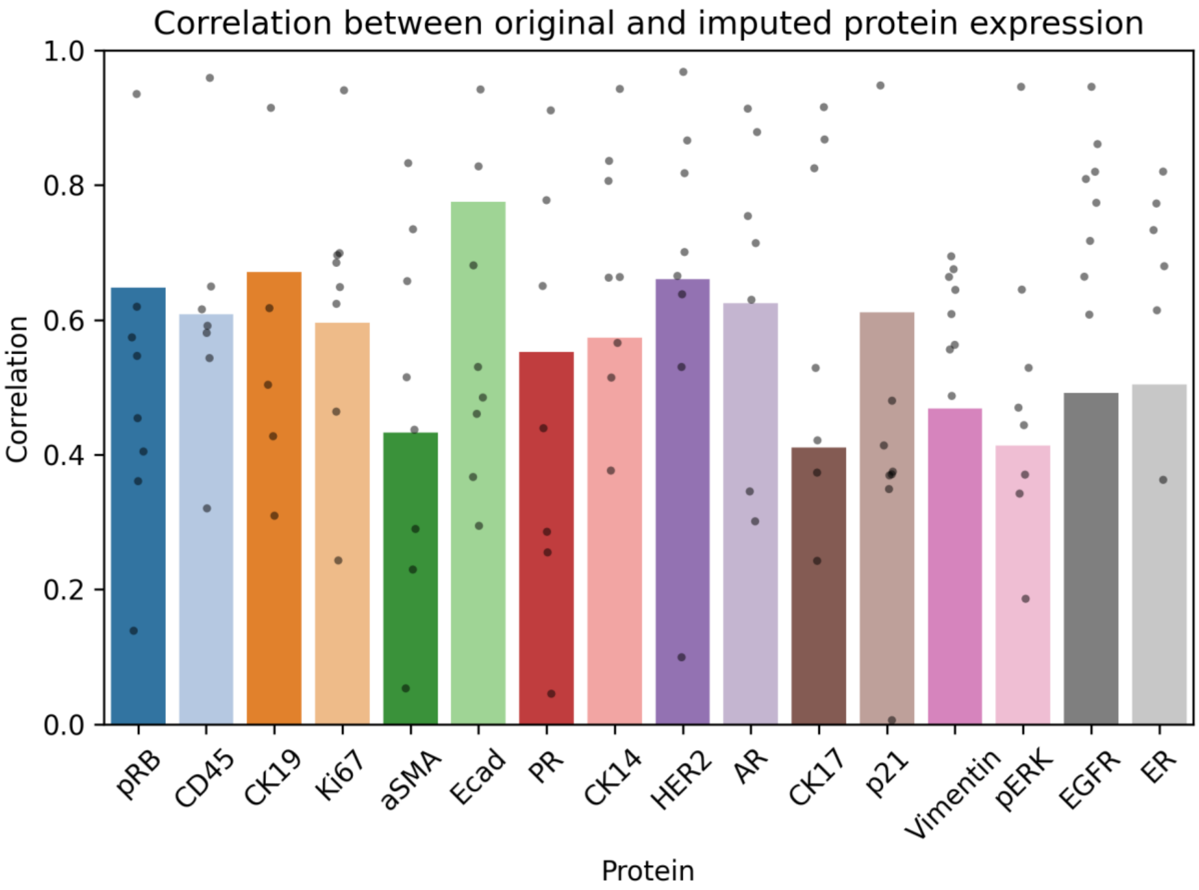

Supplementary Figure 2: Calculated correlation coefficient between original protein values and imputed protein values, show high correlation for almost all proteins. The highest correlation can be observed for EGFR with a value ≥ 0.75 indicating very strong relation. Notable exceptions are CK19 and PR with a correlation coefficient of about ≥ 0.4, indicating moderate relation. Source data is provided as a Source Data file.

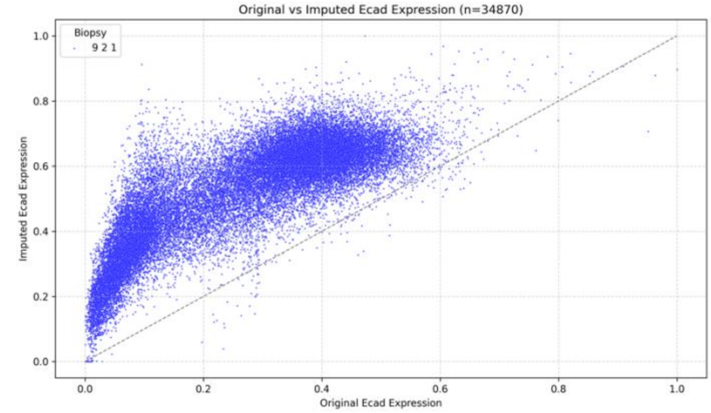
Original vs Imputed Ecad Expression (n=34870)
Biopsy
9 2 1
Imputed Ecad Expression
Original Ecad Expression

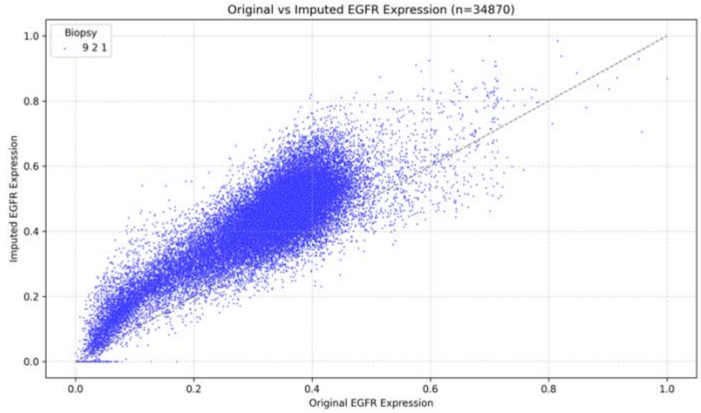
Original vs Imputed EGFR Expression (n=34870)
Biopsy
9 2 1
Imputed EGFR Expression
Original EGFR Expression

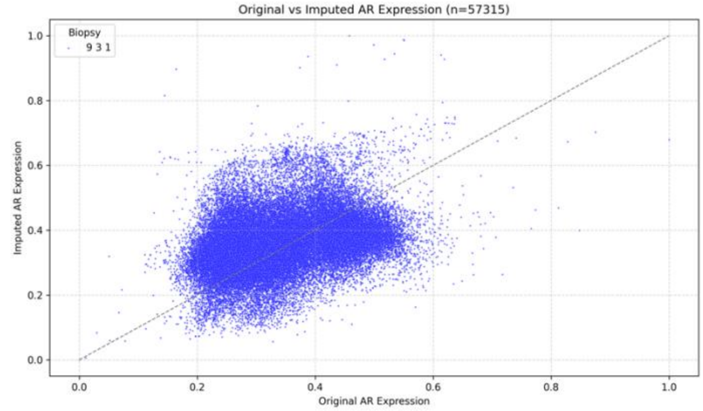
Original vs Imputed AR Expression (n=57315)
Biopsy
9 3 1
Imputed AR Expression
Original AR Expression

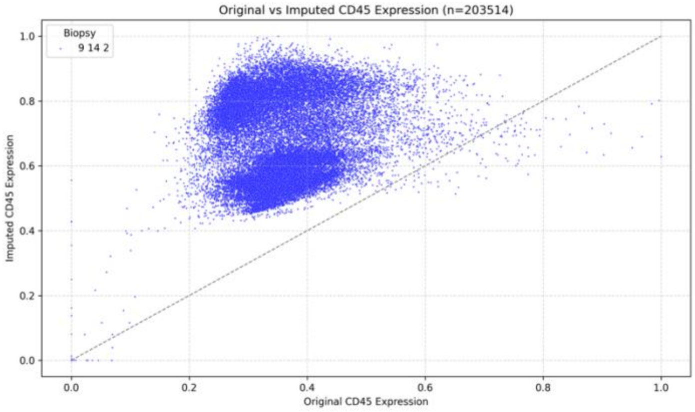
Original vs Imputed CD45 Expression (n=203514)
Biopsy
9 14 2
Imputed CD45 Expression
Original CD45 Expression

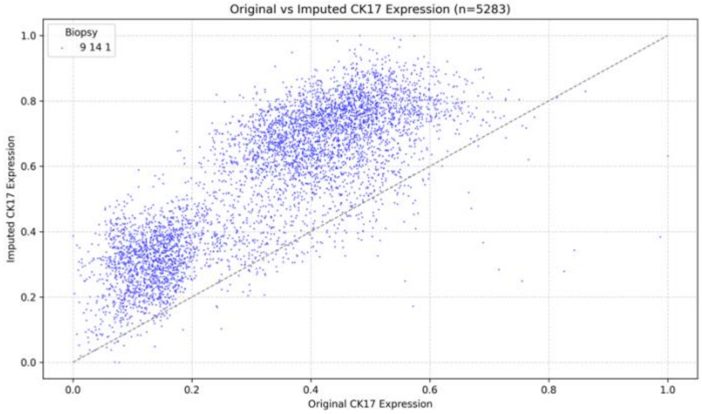
Original vs Imputed CK17 Expression (n=5283)
Biopsy
9 14 1
Imputed CK17 Expression
Original CK17 Expression

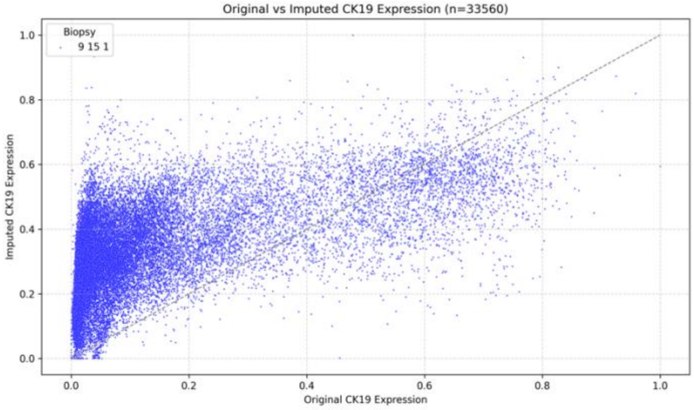
Original vs Imputed CK19 Expression (n=33560)
Biopsy
9 15 1
Imputed CK19 Expression
Original CK19 Expression

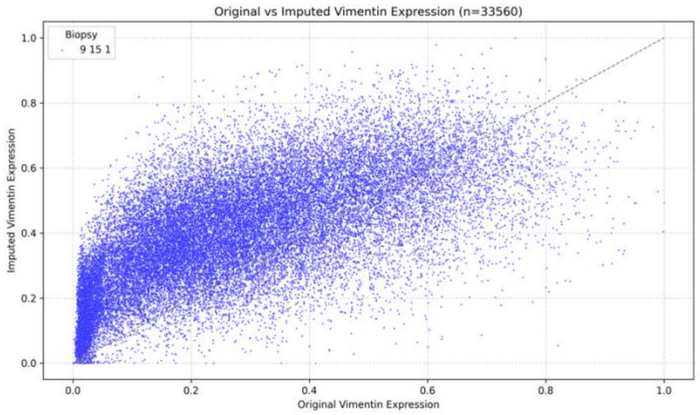
Original vs Imputed Vimentin Expression (n=33560)
Biopsy
9 15 1
Imputed Vimentin Expression
Original Vimentin Expression

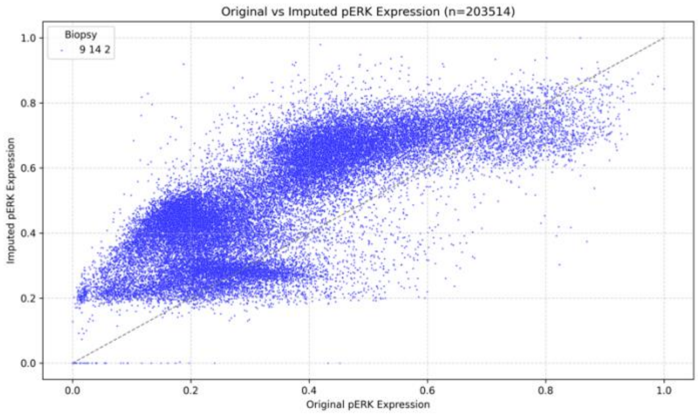
Original vs Imputed pERK Expression (n=203514)
Biopsy
9 14 2
Imputed pERK Expression
Original pERK Expression

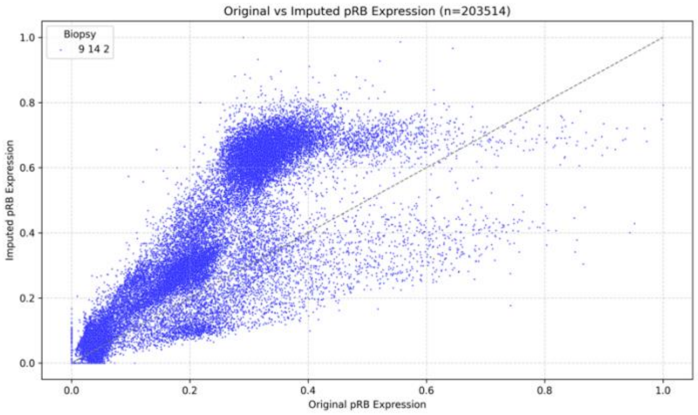
Original vs Imputed pRB Expression (n=203514)
Biopsy
9 14 2
Imputed pRB Expression
Original pRB Expression

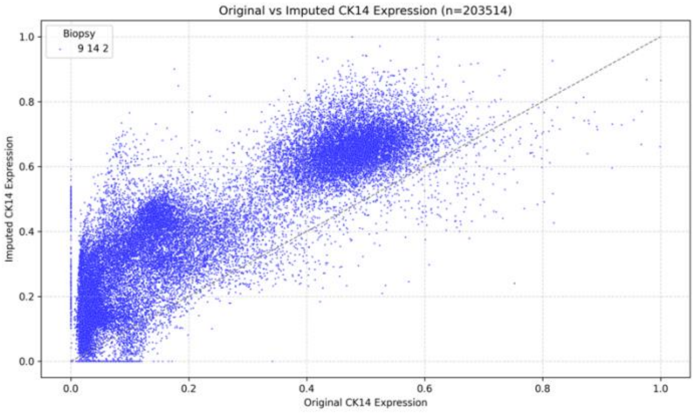
Original vs Imputed CK14 Expression (n=203514)
Biopsy
9 14 2
Imputed CK14 Expression
Original CK14 Expression

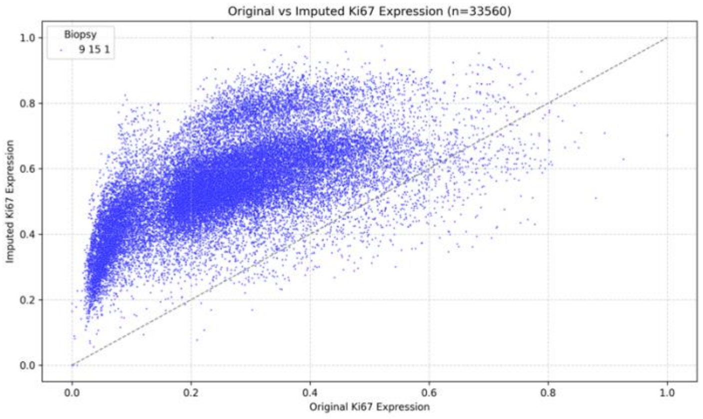
Original vs Imputed Ki67 Expression (n=33560)
Biopsy
9 15 1
Imputed Ki67 Expression
Original Ki67 Expression

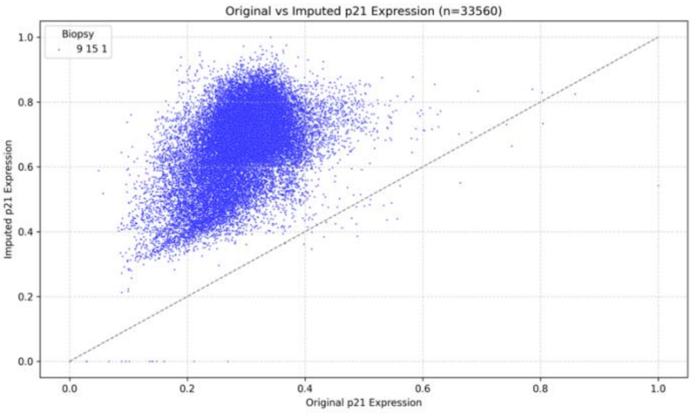
Original vs Imputed p21 Expression (n=33560)
Biopsy
9 15 1
Imputed p21 Expression
Original p21 Expression

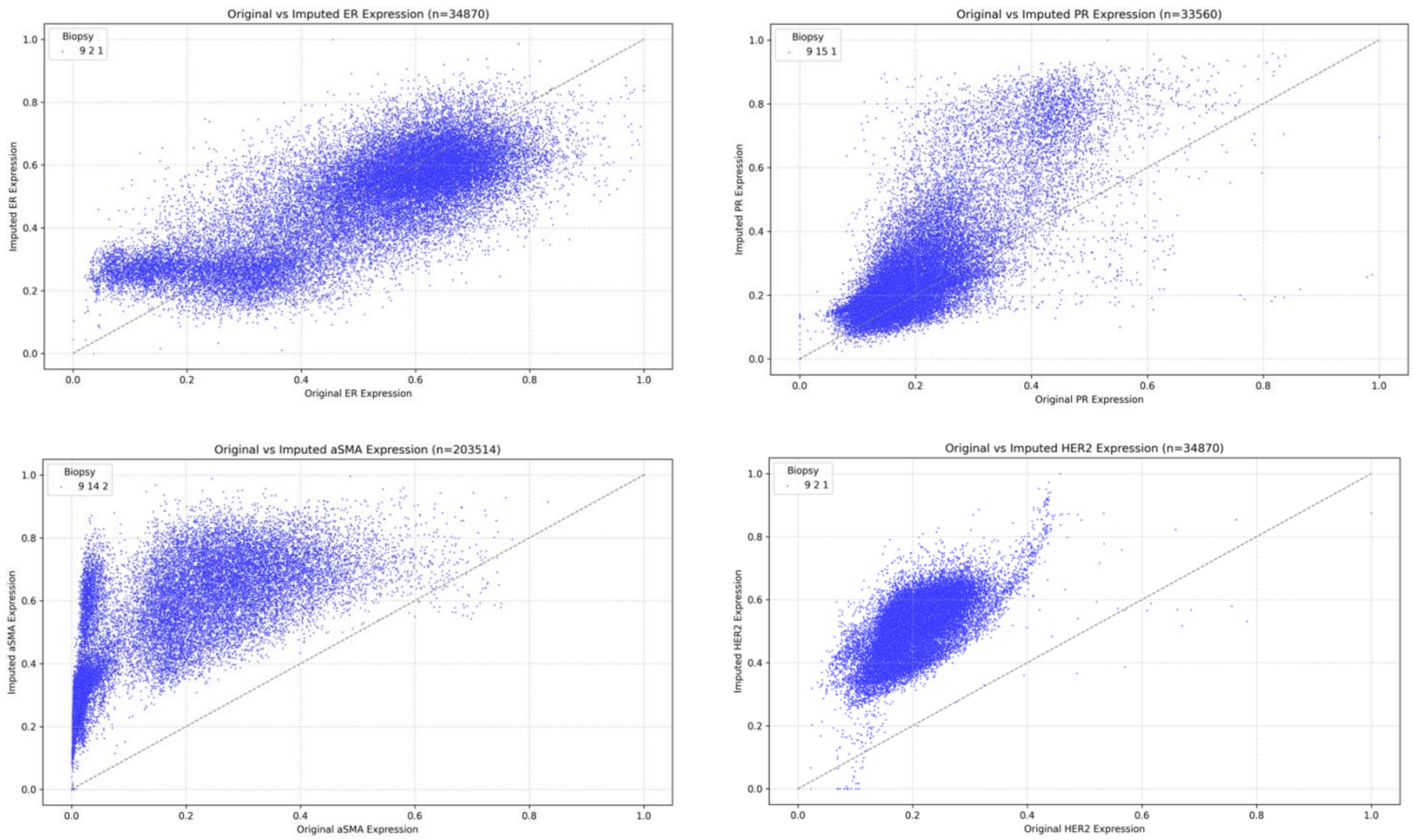

Supplementary Figure 3: Representative scatter plots illustrating the relationship between ground truth and imputed data across all proteins demonstrate moderate to strong correlation. The red line represents the theoretical line of perfect agreement between original and imputed values. Source data is provided as a Source Data file.

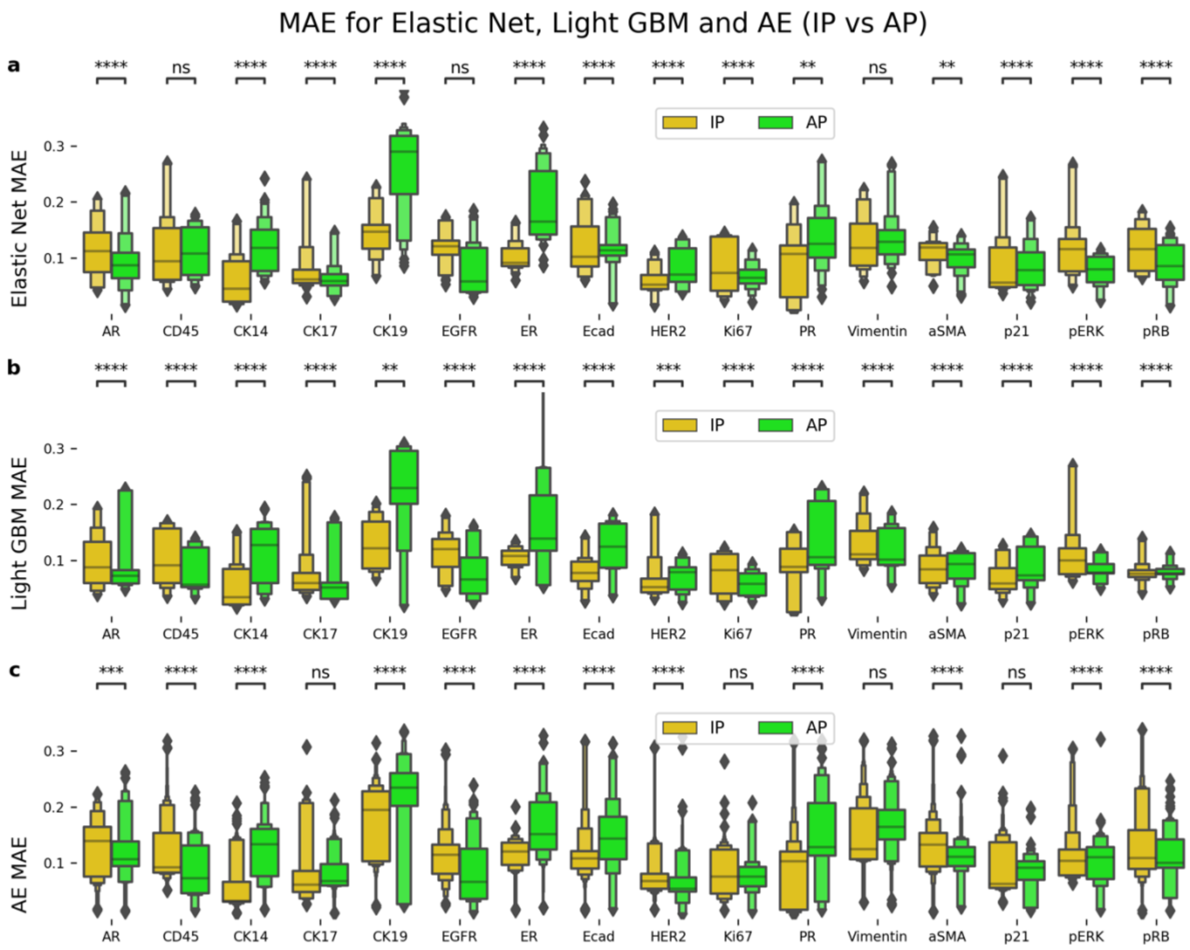

Supplementary Figure 4: Performance comparison between In Patient (IP) and Across Patient (AP): In Patient (IP, golden) is compared to Across Patient (AP, green) across models: a) Elastic Net (top), b) Light GBM (middle) and c) Autoencoder (AE, bottom). For means of comparison the Mean Absolute Error (MAE) was calculated. Statistical analysis using Mann-Whittney-Wilcoxon and multi-hypothesis testing using Benjamini-Hochberg correction. Source data are provided as a Source Data file. Supplementary Table 17, Supplementary Table 18 and Supplementary Table 19 provide detailed description for all boxen plots. Each boxenplot displays nested boxes corresponding to progressively smaller quantile ranges. The central, widest box represents the interquartile range (25th–75th percentiles), capturing the middle 50% of the data. Narrower boxes above and below reflect increasingly extreme quantiles (e.g., 12.5th–87.5th, 6.25th–93.75th), providing a detailed view of distribution tails. Outliers beyond the outermost quantile range are shown as diamonds.

p-values:

ns: not significant, $p \leq 1.00e+00$

*: $1.00e-02 < p \leq 5.00e-02$

**: $1.00e-03 < p \leq 1.00e-02$

***: $1.00e-04 < p \leq 1.00e-03$

****: $p \leq 1.00e-04$

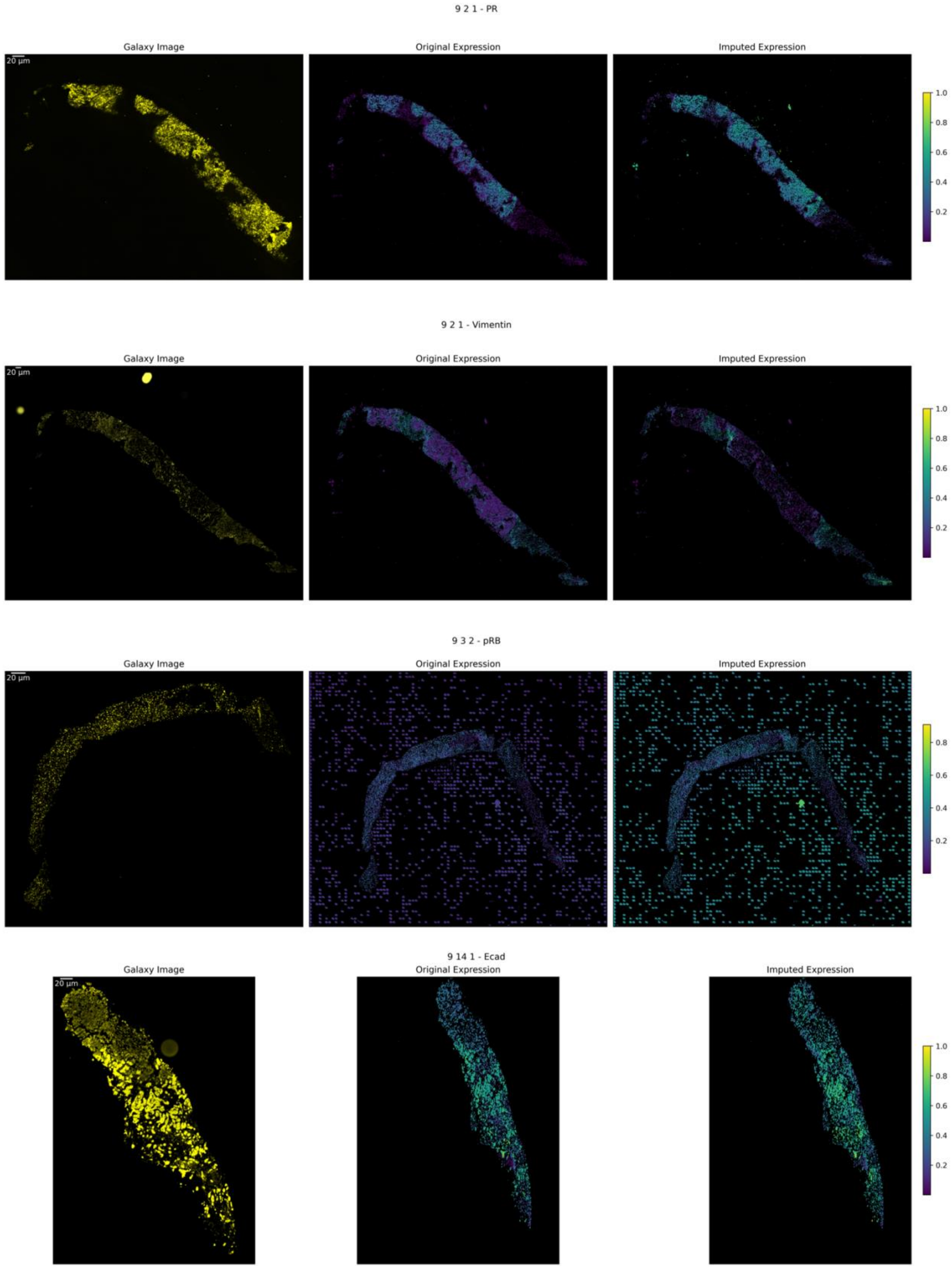
9 2 1 - PR
Galaxy Image
Original Expression
Imputed Expression
20 µm
1.0
0.8
0.6
0.4
0.2
9 2 1 - Vimentin
Galaxy Image
Original Expression
Imputed Expression
20 µm
1.0
0.8
0.6
0.4
0.2
9 3 2 - pRB
Galaxy Image
Original Expression
Imputed Expression
20 µm
0.8
0.6
0.4
0.2
9 14 1 - Ecad
Galaxy Image
Original Expression
Imputed Expression
20 µm
1.0
0.8
0.6
0.4
0.2

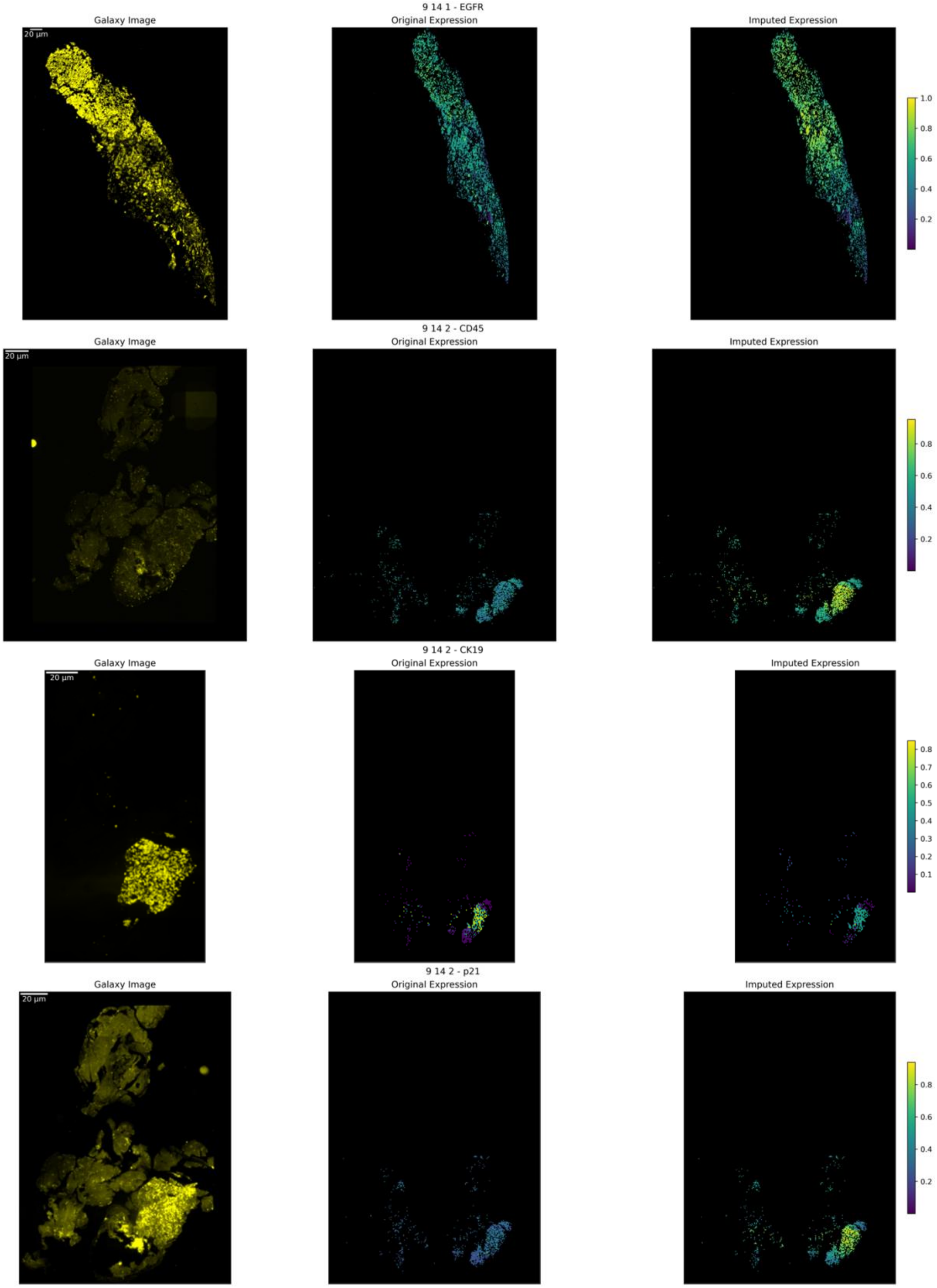
9 14 1 - EGFR
Galaxy Image
Original Expression
Imputed Expression
20 µm
1.0
0.8
0.6
0.4
0.2
9 14 2 - CD45
Galaxy Image
Original Expression
Imputed Expression
20 µm
0.8
0.6
0.4
0.2
9 14 2 - CK19
Galaxy Image
Original Expression
Imputed Expression
20 µm
0.8
0.7
0.6
0.5
0.4
0.3
0.2
0.1
9 14 2 - p21
Galaxy Image
Original Expression
Imputed Expression
20 µm
0.8
0.6
0.4
0.2

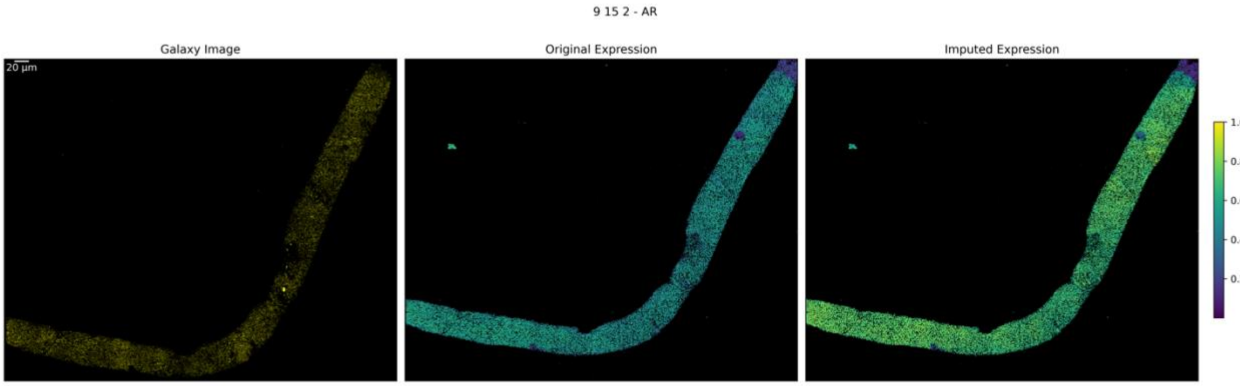
9 15 2 - AR
Galaxy Image
Original Expression
Imputed Expression
20 µm
1.0
0.8
0.6
0.4
0.2

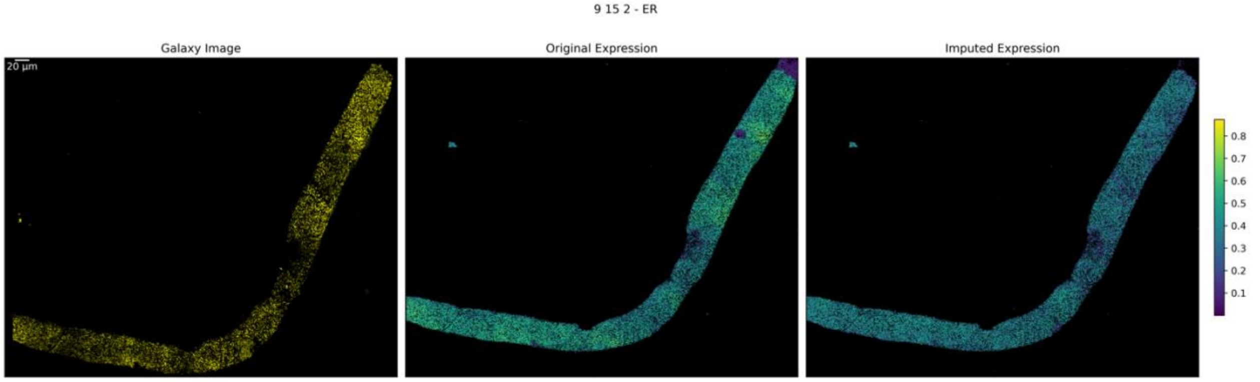
9 15 2 - ER
Galaxy Image
Original Expression
Imputed Expression
20 µm
0.8
0.7
0.6
0.5
0.4
0.3
0.2
0.1

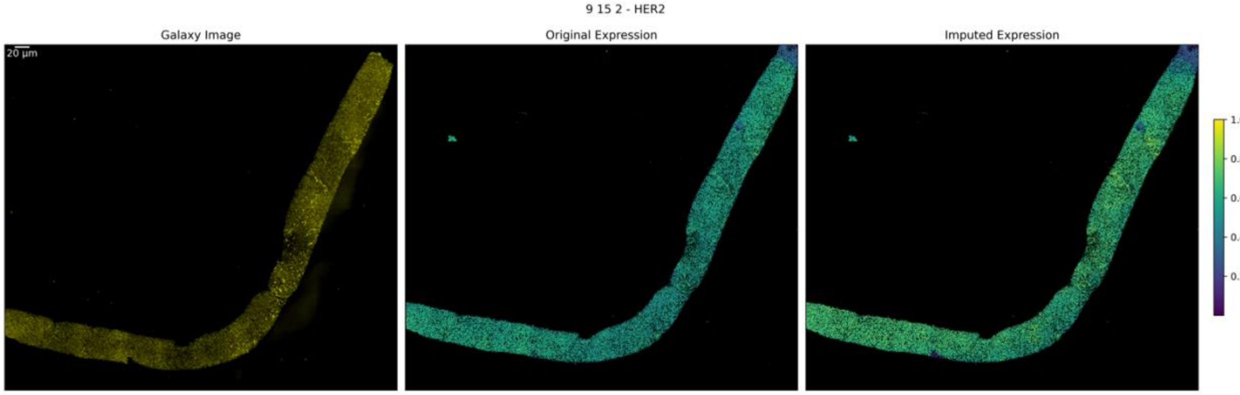
9 15 2 - HER2
Galaxy Image
Original Expression
Imputed Expression
20 µm
1.0
0.8
0.6
0.4
0.2

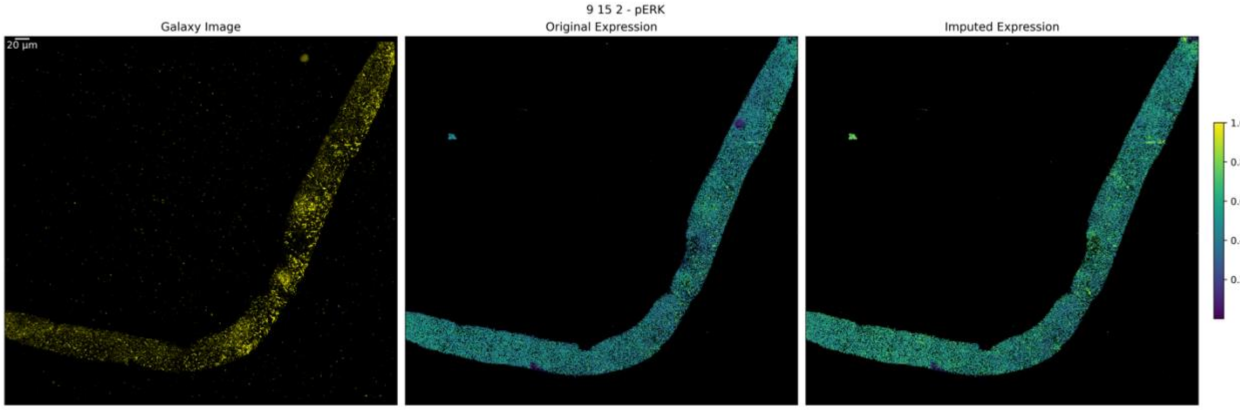
9 15 2 - pERK
Galaxy Image
Original Expression
Imputed Expression
20 µm
1.0
0.8
0.6
0.4
0.2

Supplementary Figure 5: A selection of protein expression is shown with in situ visualization, alongside the original and imputed protein expression data. The imputed protein expression successfully mirrors the overall structural patterns of the original data.

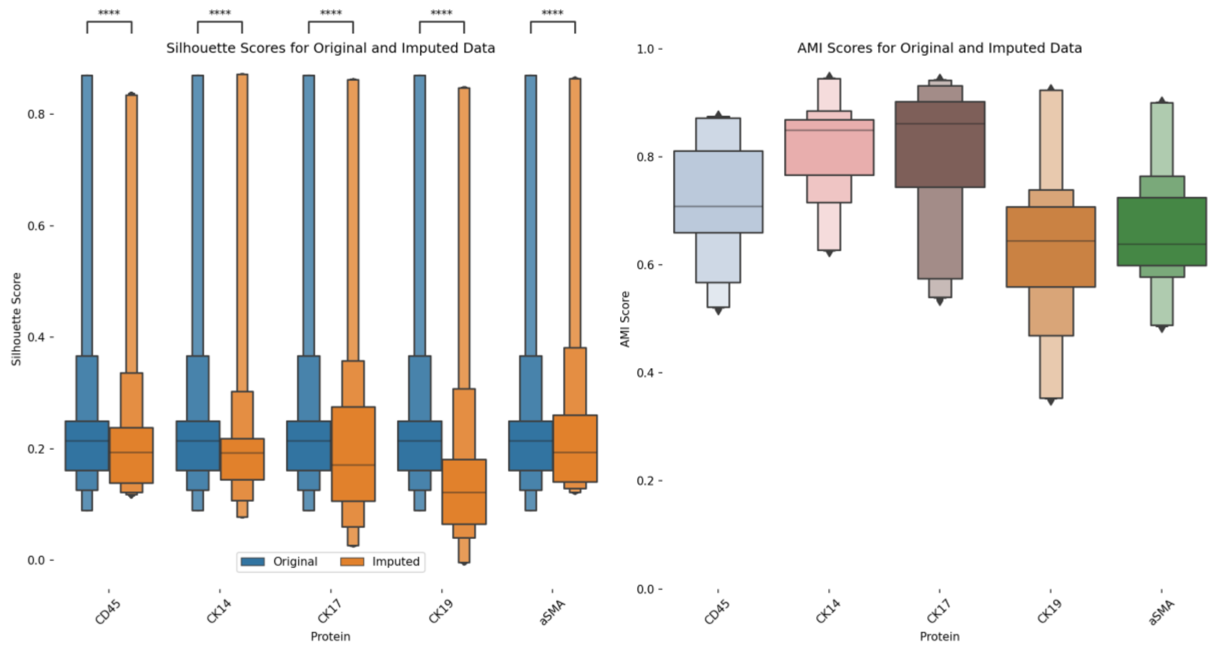

Supplementary Figure 6: Silhouette scores indicate a slight decrease in clustering performance for the proteins involved in phenotype calling, with differences ranging from 0.01 (CD45) to 0.05 (CK19). AMI scores reveal substantial information overlap between clusters of original and imputed data for these proteins, with the highest score exceeding 0.8 for CK14 and CK17, and the lowest score remaining above 0.6 for CK19. Source data are provided as a Source Data file. Supplementary Table 20, Supplementary Table 21 provide detailed boxenplot descriptions for this figure. Each boxenplot displays nested boxes corresponding to progressively smaller quantile ranges. The central, widest box represents the interquartile range (25th–75th percentiles), capturing the middle 50% of the data. Narrower boxes above and below reflect increasingly extreme quantiles (e.g., 12.5th–87.5th, 6.25th–93.75th), providing a detailed view of distribution tails. Outliers beyond the outermost quantile range are shown as diamonds.

p-values:
ns: not significant, p ≤ 1.00e+00
*: 1.00e-02 < p ≤ 5.00e-02
**: 1.00e-03 < p ≤ 1.00e-02
***: 1.00e-04 < p ≤ 1.00e-03
****: p ≤ 1.00e-04

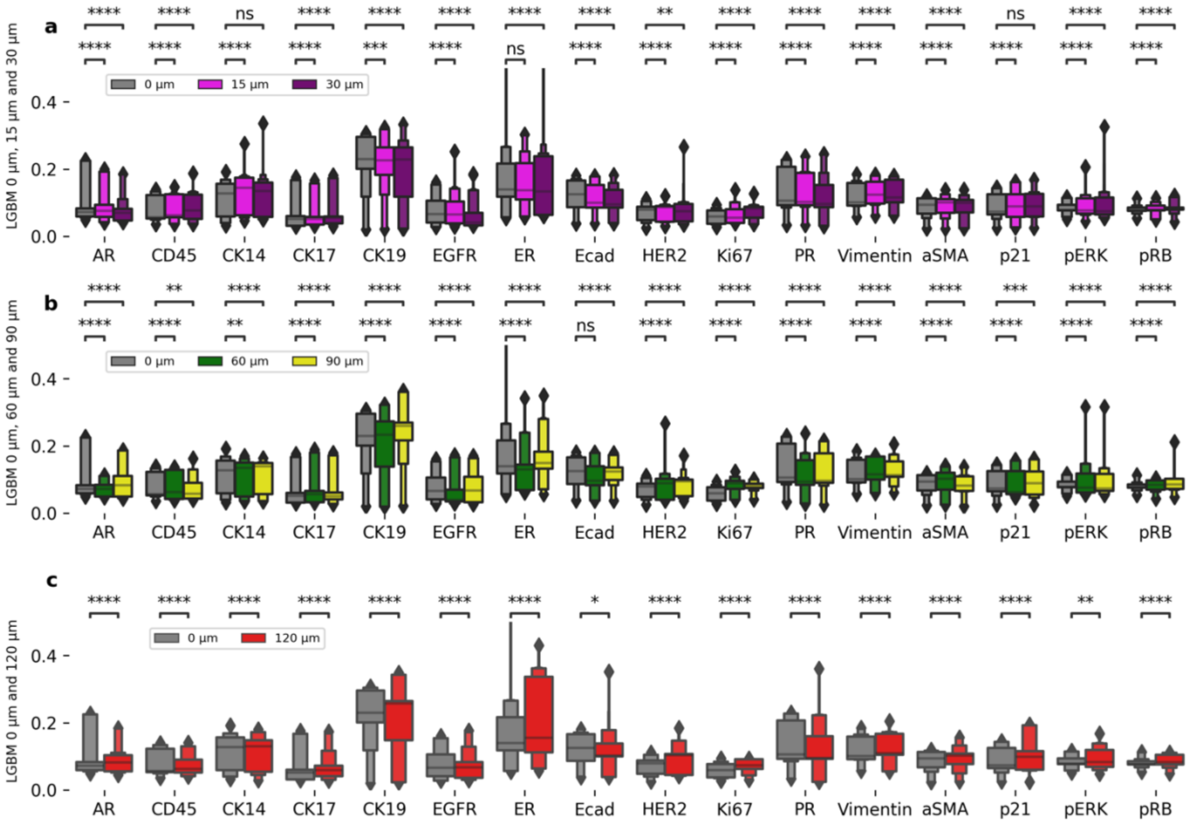

Supplementary Figure 7: Performance comparison between baseline (0 µm) and increasing spatial distances of 15, 30, 60, 90 and 120 µm. Light GBM results comparing increasing spatial distances to the baseline (0 µm in grey) across proteins. **a)** Baseline compared to 15 µm (magenta) and 30 µm (plum). **b)** Baseline compared to 60 µm (green) and 90 µm (yellow) and **c)** Baseline compared to 120 µm (red). For means of comparison the Mean Absolute Error (MAE) was calculated. Statistical analysis using Mann-Whittney-Wilcoxon and multi-hypothesis testing using Benjamini-Hochberg correction. Source data are provided as a Source Data file. Supplementary Table 22 provides detailed descriptions for all boxen plots. Each boxenplot displays nested boxes corresponding to progressively smaller quantile ranges. The central, widest box represents the interquartile range (25th–75th percentiles), capturing the middle 50% of the data. Narrower boxes above and below reflect increasingly extreme quantiles (e.g., 12.5th–87.5th, 6.25th–93.75th), providing a detailed view of distribution tails. Outliers beyond the outermost quantile range are shown as diamonds.

p-values:

ns: not significant, p ≤ 1.00e+00

*: 1.00e-02 < p ≤ 5.00e-02

**: 1.00e-03 < p ≤ 1.00e-02

***: 1.00e-04 < p ≤ 1.00e-03

****: p ≤ 1.00e-04

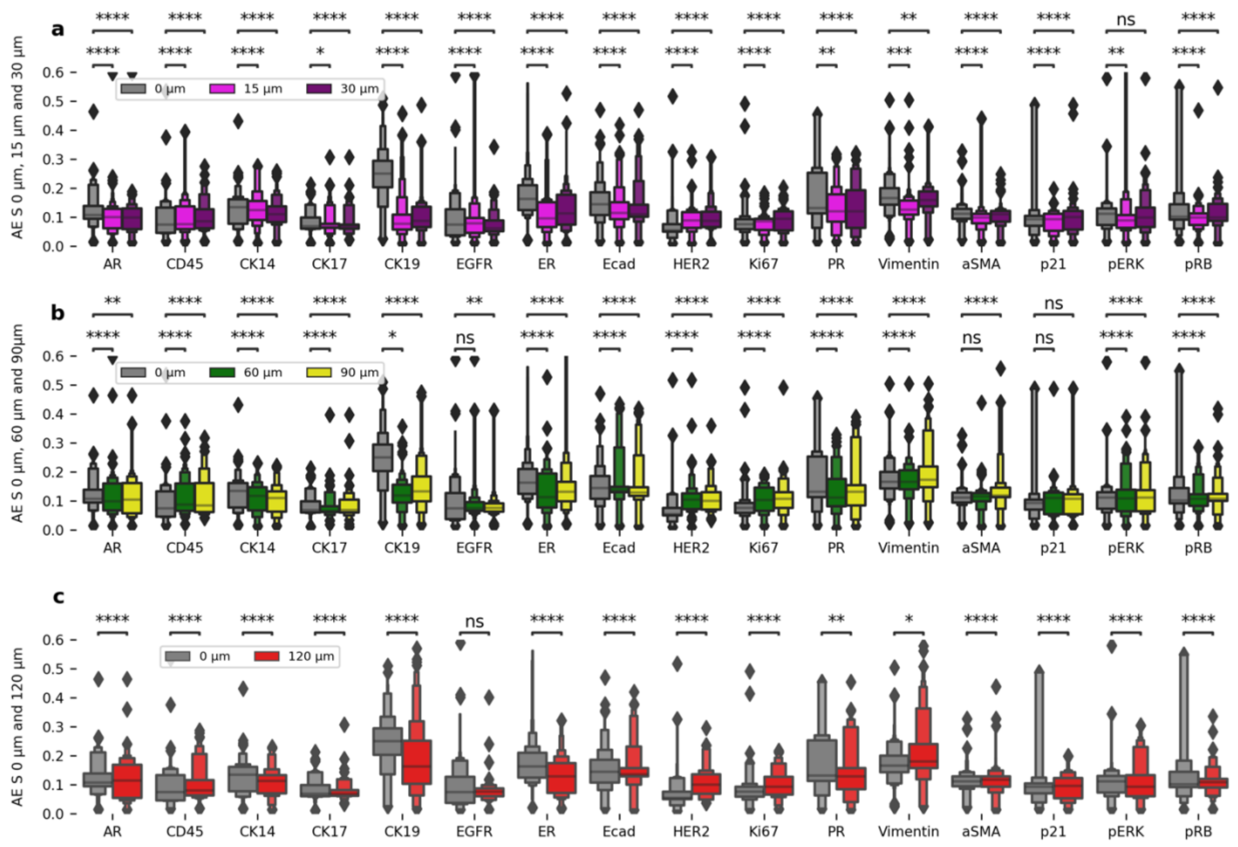

Supplementary Figure 8: Performance comparison between baseline (0 µm) and increasing spatial distances of 15, 30, 60, 90 and 120 µm. Autoencoder (AE) results comparing increasing spatial distances to the baseline (0 µm in grey) across proteins. **a)** Baseline compared to 15 µm (magenta) and 30 µm (plum). **b)** Baseline compared to 60 µm (green) and 90 µm (yellow) and **c)** Baseline compared to 120 µm (red). For means of comparison the Mean Absolute Error (MAE) was calculated. Statistical analysis using Mann-Whittney-Wilcoxon and multi-hypothesis testing using Benjamini-Hochberg correction. Source data are provided as a Source Data file. Supplementary Table 23 provides detailed descriptions for all boxen plots. Each boxenplot displays nested boxes corresponding to progressively smaller quantile ranges. The central, widest box represents the interquartile range (25th–75th percentiles), capturing the middle 50% of the data. Narrower boxes above and below reflect increasingly extreme quantiles (e.g., 12.5th–87.5th, 6.25th–93.75th), providing a detailed view of distribution tails. Outliers beyond the outermost quantile range are shown as diamonds.

p-values:

ns: not significant, $p \leq 1.00e+00$

*: $1.00e-02 < p \leq 5.00e-02$

**: $1.00e-03 < p \leq 1.00e-02$

***: $1.00e-04 < p \leq 1.00e-03$

****: $p \leq 1.00e-04$

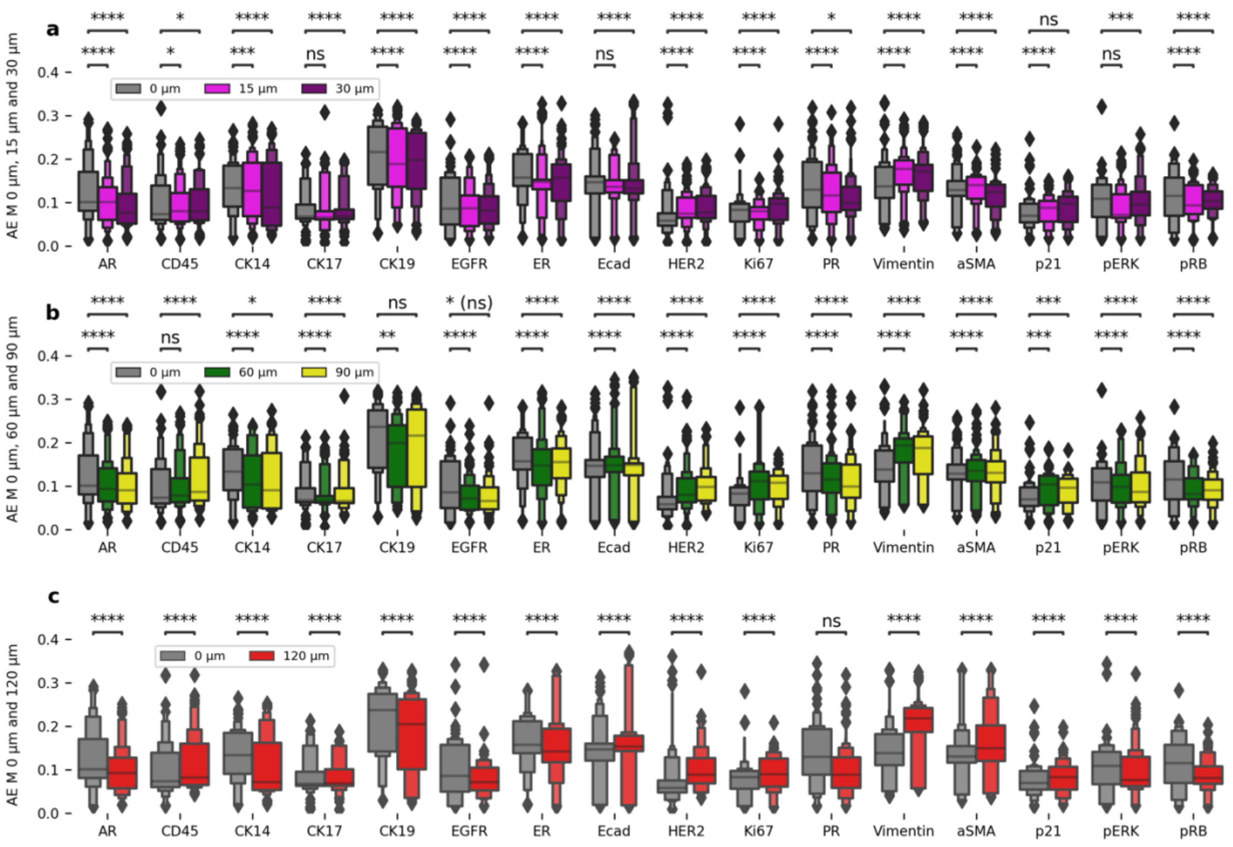

Supplementary Figure 9: Performance comparison between baseline (0 µm) and increasing distances of 15, 30, 60, 90 and 120 µm. Autoencoder M (AE M) results using multi protein imputation comparing increasing spatial distances to the baseline (0 µm in grey) across proteins. **a)** Baseline compared to 15 µm (magenta) and 30 µm (plum). **b)** Baseline compared to 60 µm (green) and 90 µm (yellow) and **c)** Baseline compared to 120 µm (red). For means of comparison the Mean Absolute Error (MAE) was calculated. Statistical analysis using Mann-Whittney-Wilcoxon and multi-hypothesis testing using Benjamini-Hochberg correction. Source data are provided as a Source Data file. Supplementary Table 24 provides detailed descriptions of all boxen plots. Each boxenplot displays nested boxes corresponding to progressively smaller quantile ranges. The central, widest box represents the interquartile range (25th–75th percentiles), capturing the middle 50% of the data. Narrower boxes above and below reflect increasingly extreme quantiles (e.g., 12.5th–87.5th, 6.25th–93.75th), providing a detailed view of distribution tails. Outliers beyond the outermost quantile range are shown as diamonds.

p-values:

ns: not significant, $p \leq 1.00e+00$

*: $1.00e-02 < p \leq 5.00e-02$

**: $1.00e-03 < p \leq 1.00e-02$

***: $1.00e-04 < p \leq 1.00e-03$

****: $p \leq 1.00e-04$

Marker	Model	Mean	Median	SEM	SD	Min	Max	Q1 (25%)	Q3 (75%)
AR	EN	0.052	0.048	0.002	0.032	0.003	0.105	0.033	0.075
AR	Null	0.128	0.079	0.007	0.109	0.03	0.362	0.053	0.158
CD45	EN	0.054	0.05	0.002	0.024	0.02	0.098	0.04	0.061
CD45	Null	0.121	0.077	0.007	0.102	0.022	0.29	0.03	0.212
CK14	EN	0.06	0.057	0.001	0.022	0.03	0.105	0.047	0.072
CK14	Null	0.102	0.054	0.007	0.105	0.02	0.346	0.026	0.14
CK17	EN	0.032	0.032	0.0	0.007	0.023	0.042	0.026	0.037
CK17	Null	0.06	0.039	0.003	0.04	0.029	0.139	0.031	0.071
CK19	EN	0.318	0.353	0.008	0.122	0.034	0.472	0.288	0.38
CK19	Null	0.426	0.345	0.015	0.236	0.187	1.0	0.299	0.466
EGFR	EN	0.024	0.018	0.001	0.02	0.001	0.057	0.011	0.03
EGFR	Null	0.101	0.086	0.005	0.074	0.028	0.231	0.031	0.141
ER	EN	0.222	0.225	0.005	0.074	0.106	0.327	0.167	0.287
ER	Null	0.295	0.285	0.011	0.163	0.036	0.563	0.184	0.422
Ecad	EN	0.078	0.067	0.003	0.044	0.019	0.155	0.043	0.113
Ecad	Null	0.16	0.151	0.004	0.069	0.068	0.254	0.101	0.226
HER2	EN	0.044	0.037	0.002	0.026	0.009	0.092	0.026	0.059
HER2	Null	0.132	0.074	0.008	0.123	0.02	0.352	0.024	0.237
Ki67	EN	0.035	0.018	0.002	0.033	0.0	0.098	0.011	0.059
Ki67	Null	0.122	0.058	0.007	0.106	0.033	0.318	0.042	0.189
Mean	EN	0.087	0.054	0.008	0.09	0.0	0.472	0.035	0.098
Mean	Null	0.165	0.12	0.013	0.151	0.02	0.999	0.05	0.247
PR	EN	0.071	0.054	0.002	0.034	0.035	0.131	0.048	0.096
PR	Null	0.093	0.072	0.003	0.049	0.049	0.201	0.054	0.119
Vimentin	EN	0.125	0.092	0.003	0.053	0.074	0.22	0.087	0.169
Vimentin	Null	0.21	0.196	0.007	0.102	0.08	0.342	0.12	0.311
aSMA	EN	0.075	0.053	0.003	0.048	0.012	0.143	0.04	0.126
aSMA	Null	0.161	0.136	0.006	0.096	0.062	0.335	0.077	0.221
p21	EN	0.042	0.039	0.001	0.022	0.02	0.095	0.027	0.044
p21	Null	0.129	0.109	0.006	0.085	0.044	0.276	0.052	0.186
pERK	EN	0.095	0.075	0.004	0.067	0.021	0.213	0.037	0.136
pERK	Null	0.252	0.12	0.013	0.207	0.086	0.605	0.094	0.394
pRB	EN	0.058	0.061	0.001	0.021	0.009	0.087	0.054	0.067
pRB	Null	0.15	0.118	0.006	0.092	0.038	0.294	0.089	0.234

Supplementary Table 3: Fig 2a Null model vs EN boxen plot description.

Marker	Model	Mean	Median	SEM	SD	Min	Max	Q1 (25%)	Q3 (75%)
AR	EN	0.093	0.087	0.003	0.055	0.016	0.216	0.063	0.109
AR	LGBM	0.088	0.072	0.0	0.055	0.049	0.227	0.059	0.083
CD45	EN	0.109	0.108	0.003	0.042	0.049	0.178	0.069	0.155
CD45	LGBM	0.075	0.057	0.0	0.034	0.036	0.139	0.053	0.123
CK14	EN	0.119	0.118	0.003	0.047	0.058	0.242	0.077	0.15
CK14	LGBM	0.108	0.127	0.0	0.05	0.032	0.193	0.059	0.156
CK17	EN	0.066	0.058	0.002	0.032	0.028	0.145	0.05	0.071
CK17	LGBM	0.062	0.052	0.0	0.046	0.029	0.177	0.032	0.06
CK19	EN	0.256	0.289	0.005	0.082	0.086	0.399	0.214	0.318
CK19	LGBM	0.211	0.229	0.001	0.088	0.02	0.308	0.201	0.296
EGFR	EN	0.079	0.058	0.003	0.047	0.03	0.185	0.039	0.117
EGFR	LGBM	0.073	0.066	0.0	0.042	0.027	0.161	0.04	0.105
ER	EN	0.192	0.165	0.004	0.061	0.087	0.332	0.141	0.255
ER	LGBM	0.151	0.139	0.001	0.075	0.054	0.589	0.117	0.217
Ecad	EN	0.112	0.113	0.003	0.041	0.019	0.196	0.104	0.123
Ecad	LGBM	0.117	0.124	0.0	0.045	0.038	0.181	0.087	0.166
HER2	EN	0.08	0.07	0.002	0.033	0.038	0.138	0.057	0.117
HER2	LGBM	0.066	0.079	0.0	0.026	0.025	0.112	0.048	0.087
Ki67	EN	0.069	0.065	0.002	0.024	0.021	0.114	0.055	0.079
Ki67	LGBM	0.059	0.058	0.0	0.022	0.027	0.093	0.037	0.077
Mean	EN	0.112	0.111	0.009	0.025	0.073	0.147	0.097	0.133
Mean	LGBM	0.098	0.1	0.009	0.026	0.053	0.128	0.085	0.116
PR	EN	0.137	0.125	0.004	0.063	0.03	0.273	0.101	0.171
PR	LGBM	0.127	0.106	0.001	0.061	0.033	0.23	0.092	0.206
Vimentin	EN	0.137	0.128	0.003	0.051	0.049	0.268	0.106	0.149
Vimentin	LGBM	0.12	0.101	0.0	0.039	0.057	0.185	0.092	0.158
aSMA	EN	0.098	0.106	0.002	0.029	0.033	0.139	0.083	0.115
aSMA	LGBM	0.085	0.094	0.0	0.031	0.023	0.12	0.067	0.113
p21	EN	0.086	0.078	0.003	0.042	0.022	0.171	0.052	0.11
p21	LGBM	0.085	0.073	0.0	0.037	0.025	0.145	0.065	0.125
pERK	EN	0.077	0.08	0.002	0.027	0.025	0.115	0.057	0.102
pERK	LGBM	0.083	0.079	0.0	0.021	0.051	0.117	0.077	0.094
pRB	EN	0.086	0.085	0.002	0.039	0.015	0.153	0.06	0.123
pRB	LGBM	0.08	0.077	0.0	0.015	0.053	0.113	0.076	0.085

Supplementary Table 4: Fig 2b EN vs LGBM boxen plot description.

Marker	Mean	Median	SEM	SD	Min	Max	Q1 (25%)	Q3 (75%)
AR	0.757	0.722	0.06	0.171	0.582	0.993	0.613	0.898
CD45	0.759	0.802	0.059	0.167	0.499	0.994	0.644	0.844
CK14	0.656	0.596	0.054	0.153	0.51	0.993	0.578	0.696
CK17	0.697	0.606	0.076	0.215	0.445	0.999	0.54	0.905
CK19	0.614	0.54	0.074	0.209	0.372	0.993	0.502	0.678
EGFR	0.725	0.655	0.062	0.175	0.494	0.993	0.624	0.855
ER	0.698	0.675	0.05	0.14	0.54	0.993	0.6	0.748
Ecad	0.692	0.686	0.068	0.192	0.495	0.993	0.509	0.83
HER2	0.692	0.589	0.066	0.186	0.516	0.993	0.565	0.862
Ki67	0.693	0.658	0.062	0.177	0.481	0.993	0.56	0.801
PR	0.633	0.573	0.065	0.184	0.443	0.993	0.549	0.651
Vimentin	0.646	0.618	0.062	0.175	0.479	0.992	0.508	0.727
aSMA	0.654	0.56	0.072	0.205	0.458	0.993	0.516	0.759
p21	0.712	0.64	0.065	0.185	0.519	0.997	0.572	0.874
pERK	0.723	0.718	0.058	0.163	0.508	0.993	0.612	0.816
pRB	0.718	0.707	0.07	0.199	0.486	0.998	0.56	0.861

Supplementary Table 5: Fig 3a ARI Score boxen plot description

Marker	Model	Mean	Median	SEM	SD	Min	Max	Q1 (25%)	Q3 (75%)
AR	Imputed	0.32	0.244	0.082	0.232	0.181	0.884	0.21	0.296
AR	Original	0.298	0.209	0.085	0.241	0.177	0.887	0.189	0.245
CD45	Imputed	0.314	0.243	0.083	0.233	0.163	0.88	0.203	0.286
CD45	Original	0.298	0.209	0.085	0.241	0.177	0.887	0.189	0.245
CK14	Imputed	0.293	0.208	0.085	0.24	0.18	0.883	0.191	0.232
CK14	Original	0.298	0.209	0.085	0.241	0.177	0.887	0.189	0.245
CK17	Imputed	0.308	0.238	0.085	0.241	0.145	0.89	0.183	0.288
CK17	Original	0.298	0.209	0.085	0.241	0.177	0.887	0.189	0.245
CK19	Imputed	0.291	0.2	0.086	0.242	0.168	0.878	0.176	0.24
CK19	Original	0.298	0.209	0.085	0.241	0.177	0.887	0.189	0.245
EGFR	Imputed	0.311	0.228	0.083	0.234	0.186	0.879	0.193	0.29
EGFR	Original	0.298	0.209	0.085	0.241	0.177	0.887	0.189	0.245
ER	Imputed	0.31	0.224	0.083	0.235	0.184	0.884	0.208	0.273
ER	Original	0.298	0.209	0.085	0.241	0.177	0.887	0.189	0.245
Ecad	Imputed	0.306	0.233	0.083	0.234	0.187	0.879	0.191	0.262
Ecad	Original	0.298	0.209	0.085	0.241	0.177	0.887	0.189	0.245
HER2	Imputed	0.308	0.235	0.083	0.235	0.182	0.881	0.2	0.262
HER2	Original	0.298	0.209	0.085	0.241	0.177	0.887	0.189	0.245
Ki67	Imputed	0.312	0.231	0.083	0.234	0.185	0.884	0.206	0.277
Ki67	Original	0.298	0.209	0.085	0.241	0.177	0.887	0.189	0.245
PR	Imputed	0.308	0.222	0.082	0.233	0.183	0.88	0.212	0.264
PR	Original	0.298	0.209	0.085	0.241	0.177	0.887	0.189	0.245
Vimentin	Imputed	0.322	0.244	0.081	0.23	0.189	0.881	0.215	0.278
Vimentin	Original	0.298	0.209	0.085	0.241	0.177	0.887	0.189	0.245
aSMA	Imputed	0.324	0.251	0.081	0.23	0.18	0.882	0.217	0.276
aSMA	Original	0.298	0.209	0.085	0.241	0.177	0.887	0.189	0.245
p21	Imputed	0.305	0.227	0.084	0.238	0.178	0.887	0.197	0.257
p21	Original	0.298	0.209	0.085	0.241	0.177	0.887	0.189	0.245
pERK	Imputed	0.32	0.248	0.082	0.232	0.178	0.886	0.222	0.274
pERK	Original	0.298	0.209	0.085	0.241	0.177	0.887	0.189	0.245
pRB	Imputed	0.3	0.197	0.085	0.242	0.181	0.888	0.192	0.256
pRB	Original	0.298	0.209	0.085	0.241	0.177	0.887	0.189	0.245

Supplementary Table 6: Fig 3b Silhouette Original vs Imputed boxen plot description.

Marker	Mean	Median	SEM	SD	Min	Max	Q1 (25%)	Q3 (75%)
CD45	0.738	0.821	0.014	0.213	0.372	0.979	0.625	0.893
CK14	0.843	0.864	0.007	0.102	0.692	0.994	0.742	0.922
CK17	0.802	0.914	0.014	0.22	0.3	0.993	0.767	0.932
CK19	0.566	0.5	0.014	0.223	0.188	0.994	0.469	0.673
aSMA	0.665	0.683	0.015	0.229	0.264	0.989	0.484	0.868

Supplementary Table 7: Fig 3c Phenotype ARI scores boxen plot description.

Marker	Mean	Median	SEM	SD	Min	Max	Q1 (25%)	Q3 (75%)
CD45	0.59	0.576	0.005	0.083	0.495	0.722	0.504	0.665
CK14	0.78	0.789	0.004	0.07	0.667	0.917	0.746	0.813
CK17	0.74	0.768	0.011	0.173	0.368	0.962	0.693	0.84
CK19	0.562	0.563	0.004	0.064	0.422	0.674	0.544	0.59
aSMA	0.629	0.64	0.004	0.06	0.488	0.71	0.611	0.67

Supplementary Table 8: Fig 3d Phenotype ARI scores boxen plot description

Marker	Model	Mean	Median	SEM	SD	Min	Max	Q1 (25%)	Q3 (75%)
AR	AE	0.13	0.107	0.002	0.057	0.014	0.464	0.094	0.138
AR	AE M	0.125	0.101	0.002	0.071	0.015	0.597	0.08	0.17
CD45	AE	0.085	0.074	0.002	0.05	0.016	0.533	0.046	0.132
CD45	AE M	0.1	0.073	0.001	0.058	0.017	0.638	0.06	0.14
CK14	AE	0.13	0.133	0.002	0.05	0.017	0.432	0.076	0.161
CK14	AE M	0.132	0.133	0.001	0.055	0.019	0.265	0.09	0.185
CK17	AE	0.086	0.068	0.001	0.034	0.01	0.212	0.06	0.098
CK17	AE M	0.084	0.068	0.001	0.039	0.008	0.212	0.063	0.095
CK19	AE	0.239	0.249	0.003	0.106	0.025	0.826	0.203	0.294
CK19	AE M	0.257	0.272	0.002	0.1	0.029	0.501	0.155	0.339
EGFR	AE	0.079	0.074	0.002	0.063	0.013	0.59	0.036	0.126
EGFR	AE M	0.122	0.086	0.003	0.126	0.016	0.591	0.049	0.158
ER	AE	0.177	0.162	0.002	0.071	0.02	0.654	0.124	0.21
ER	AE M	0.183	0.159	0.002	0.083	0.019	0.722	0.139	0.219
Ecad	AE	0.146	0.144	0.002	0.075	0.019	0.47	0.107	0.185
Ecad	AE M	0.144	0.147	0.002	0.065	0.017	0.47	0.121	0.16
HER2	AE	0.063	0.054	0.001	0.045	0.01	0.706	0.049	0.075
HER2	AE M	0.07	0.058	0.001	0.047	0.009	0.706	0.048	0.075
Ki67	AE	0.08	0.076	0.001	0.043	0.014	0.491	0.058	0.093
Ki67	AE M	0.093	0.083	0.002	0.083	0.014	0.416	0.056	0.098
Mean	AE	0.128	0.127	0.016	0.045	0.055	0.202	0.101	0.156
Mean	AE M	0.13	0.133	0.013	0.037	0.058	0.174	0.114	0.154
PR	AE	0.182	0.131	0.004	0.118	0.017	0.455	0.113	0.253
PR	AE M	0.134	0.13	0.001	0.062	0.017	0.601	0.089	0.194
Vimentin	AE	0.171	0.165	0.002	0.052	0.025	0.505	0.143	0.199
Vimentin	AE M	0.181	0.171	0.002	0.1	0.039	0.505	0.114	0.197
aSMA	AE	0.105	0.111	0.001	0.038	0.022	0.327	0.095	0.129
aSMA	AE M	0.138	0.13	0.001	0.063	0.029	0.438	0.116	0.154
p21	AE	0.122	0.092	0.004	0.125	0.016	0.487	0.07	0.104
p21	AE M	0.073	0.069	0.001	0.03	0.016	0.487	0.054	0.097
pERK	AE	0.103	0.112	0.002	0.053	0.017	0.65	0.072	0.129
pERK	AE M	0.102	0.109	0.001	0.051	0.017	0.65	0.067	0.141
pRB	AE	0.155	0.102	0.004	0.138	0.015	0.549	0.092	0.144
pRB	AE M	0.131	0.115	0.002	0.097	0.016	0.47	0.075	0.159

Supplementary Table 9: Fig 4b AE vs AE Multi performance boxen plot description

Marker	Model	Mean	Median	SEM	SD	Min	Max	Q1 (25%)	Q3 (75%)
AR	AE	0.13	0.107	0.002	0.057	0.014	0.464	0.094	0.138
AR	AE M	0.125	0.101	0.002	0.071	0.015	0.597	0.08	0.17
AR	EN	0.093	0.087	0.003	0.055	0.016	0.216	0.063	0.109
AR	LGBM	0.088	0.072	0.0	0.055	0.049	0.227	0.059	0.083
CD45	AE	0.085	0.074	0.002	0.05	0.016	0.533	0.046	0.132
CD45	AE M	0.1	0.073	0.001	0.058	0.017	0.638	0.06	0.14
CD45	EN	0.109	0.108	0.003	0.042	0.049	0.178	0.069	0.155
CD45	LGBM	0.075	0.057	0.0	0.034	0.036	0.139	0.053	0.123
CK14	AE	0.13	0.133	0.002	0.05	0.017	0.432	0.076	0.161
CK14	AE M	0.132	0.133	0.001	0.055	0.019	0.265	0.09	0.185
CK14	EN	0.119	0.118	0.003	0.047	0.058	0.242	0.077	0.15
CK14	LGBM	0.108	0.127	0.0	0.05	0.032	0.193	0.059	0.156
CK17	AE	0.086	0.068	0.001	0.034	0.01	0.212	0.06	0.098
CK17	AE M	0.084	0.068	0.001	0.039	0.008	0.212	0.063	0.095
CK17	EN	0.066	0.058	0.002	0.032	0.028	0.145	0.05	0.071
CK17	LGBM	0.062	0.052	0.0	0.046	0.029	0.177	0.032	0.06
CK19	AE	0.239	0.249	0.003	0.106	0.025	0.826	0.203	0.294
CK19	AE M	0.257	0.272	0.002	0.1	0.029	0.501	0.155	0.339
CK19	EN	0.256	0.289	0.005	0.082	0.086	0.399	0.214	0.318
CK19	LGBM	0.211	0.229	0.001	0.088	0.02	0.308	0.201	0.296
EGFR	AE	0.079	0.074	0.002	0.063	0.013	0.59	0.036	0.126
EGFR	AE M	0.122	0.086	0.003	0.126	0.016	0.591	0.049	0.158
EGFR	EN	0.079	0.058	0.003	0.047	0.03	0.185	0.039	0.117
EGFR	LGBM	0.073	0.066	0.0	0.042	0.027	0.161	0.04	0.105
ER	AE	0.177	0.162	0.002	0.071	0.02	0.654	0.124	0.21
ER	AE M	0.183	0.159	0.002	0.083	0.019	0.722	0.139	0.219
ER	EN	0.192	0.165	0.004	0.061	0.087	0.332	0.141	0.255
ER	LGBM	0.151	0.139	0.001	0.075	0.054	0.589	0.117	0.217
Ecad	AE	0.146	0.144	0.002	0.075	0.019	0.47	0.107	0.185
Ecad	AE M	0.144	0.147	0.002	0.065	0.017	0.47	0.121	0.16
Ecad	EN	0.112	0.113	0.003	0.041	0.019	0.196	0.104	0.123
Ecad	LGBM	0.117	0.124	0.0	0.045	0.038	0.181	0.087	0.166
HER2	AE	0.063	0.054	0.001	0.045	0.01	0.706	0.049	0.075
HER2	AE M	0.07	0.058	0.001	0.047	0.009	0.706	0.048	0.075
HER2	EN	0.08	0.07	0.002	0.033	0.038	0.138	0.057	0.117
HER2	LGBM	0.066	0.079	0.0	0.026	0.025	0.112	0.048	0.087
Ki67	AE	0.08	0.076	0.001	0.043	0.014	0.491	0.058	0.093
Ki67	AE M	0.093	0.083	0.002	0.083	0.014	0.416	0.056	0.098
Ki67	EN	0.069	0.065	0.002	0.024	0.021	0.114	0.055	0.079
Ki67	LGBM	0.059	0.058	0.0	0.022	0.027	0.093	0.037	0.077
Mean	AE	0.128	0.127	0.016	0.045	0.055	0.202	0.101	0.156
Mean	AE M	0.13	0.133	0.013	0.037	0.058	0.174	0.114	0.154

PR	AE	0.182	0.131	0.004	0.118	0.017	0.455	0.113	0.253
PR	AE M	0.134	0.13	0.001	0.062	0.017	0.601	0.089	0.194
PR	EN	0.137	0.125	0.004	0.063	0.03	0.273	0.101	0.171
PR	LGBM	0.127	0.106	0.001	0.061	0.033	0.23	0.092	0.206
Vimentin	AE	0.171	0.165	0.002	0.052	0.025	0.505	0.143	0.199
Vimentin	AE M	0.181	0.171	0.002	0.1	0.039	0.505	0.114	0.197
Vimentin	EN	0.137	0.128	0.003	0.051	0.049	0.268	0.106	0.149
Vimentin	LGBM	0.12	0.101	0.0	0.039	0.057	0.185	0.092	0.158
aSMA	AE	0.105	0.111	0.001	0.038	0.022	0.327	0.095	0.129
aSMA	AE M	0.138	0.13	0.001	0.063	0.029	0.438	0.116	0.154
aSMA	EN	0.098	0.106	0.002	0.029	0.033	0.139	0.083	0.115
aSMA	LGBM	0.085	0.094	0.0	0.031	0.023	0.12	0.067	0.113
p21	AE	0.122	0.092	0.004	0.125	0.016	0.487	0.07	0.104
p21	AE M	0.073	0.069	0.001	0.03	0.016	0.487	0.054	0.097
p21	EN	0.086	0.078	0.003	0.042	0.022	0.171	0.052	0.11
p21	LGBM	0.085	0.073	0.0	0.037	0.025	0.145	0.065	0.125
pERK	AE	0.103	0.112	0.002	0.053	0.017	0.65	0.072	0.129
pERK	AE M	0.102	0.109	0.001	0.051	0.017	0.65	0.067	0.141
pERK	EN	0.077	0.08	0.002	0.027	0.025	0.115	0.057	0.102
pERK	LGBM	0.083	0.079	0.0	0.021	0.051	0.117	0.077	0.094
pRB	AE	0.155	0.102	0.004	0.138	0.015	0.549	0.092	0.144
pRB	AE M	0.131	0.115	0.002	0.097	0.016	0.47	0.075	0.159
pRB	EN	0.086	0.085	0.002	0.039	0.015	0.153	0.06	0.123
pRB	LGBM	0.08	0.077	0.0	0.015	0.053	0.113	0.076	0.085

Supplementary Table 10: Fig 4c EN vs LGBM vs AE vs AE M boxenplot description.

Marker	Model	Mean	Median	SEM	SD	Min	Max	Q1 (25%)	Q3 (75%)
AR	AE	0.239	0.208	0.005	0.168	0.001	0.621	0.111	0.327
AR	EN	0.038	0.03	0.001	0.031	0.0	0.112	0.012	0.054
AR	LGBM	0.004	0.003	0.0	0.003	0.0	0.019	0.001	0.005
CD45	AE	0.292	0.325	0.004	0.131	0.034	0.559	0.181	0.378
CD45	EN	0.037	0.034	0.001	0.023	0.004	0.086	0.018	0.057
CD45	LGBM	0.015	0.011	0.0	0.014	0.003	0.081	0.008	0.015
CK14	AE	0.142	0.116	0.003	0.101	0.013	0.402	0.062	0.182
CK14	EN	0.03	0.021	0.001	0.026	0.002	0.091	0.007	0.048
CK14	LGBM	0.011	0.005	0.001	0.017	0.001	0.098	0.002	0.011
CK17	AE	0.27	0.235	0.004	0.127	0.097	0.684	0.183	0.314
CK17	EN	0.048	0.034	0.001	0.038	0.005	0.146	0.015	0.073
CK17	LGBM	0.083	0.028	0.005	0.173	0.008	1.0	0.017	0.063

CK19	AE	0.434	0.413	0.004	0.122	0.198	0.754	0.358	0.501
CK19	EN	0.367	0.284	0.006	0.208	0.053	1.0	0.231	0.526
CK19	LGBM	0.205	0.189	0.003	0.1	0.059	0.626	0.154	0.244
EGFR	AE	0.222	0.18	0.005	0.157	0.032	0.575	0.098	0.339
EGFR	EN	0.055	0.056	0.001	0.044	0.002	0.151	0.012	0.084
EGFR	LGBM	0.005	0.003	0.0	0.005	0.0	0.034	0.002	0.006
ER	AE	0.172	0.151	0.002	0.074	0.05	0.354	0.11	0.23
ER	EN	0.041	0.036	0.001	0.024	0.01	0.107	0.019	0.057
ER	LGBM	0.064	0.05	0.002	0.053	0.015	0.254	0.031	0.069
Ecad	AE	0.175	0.146	0.003	0.09	0.038	0.335	0.105	0.273
Ecad	EN	0.05	0.038	0.001	0.035	0.006	0.181	0.028	0.06
Ecad	LGBM	0.037	0.027	0.001	0.023	0.007	0.106	0.021	0.051
HER2	AE	0.167	0.163	0.003	0.104	0.0	0.423	0.071	0.243
HER2	EN	0.034	0.023	0.001	0.032	0.0	0.102	0.008	0.059
HER2	LGBM	0.022	0.013	0.001	0.025	0.003	0.147	0.01	0.024
Ki67	AE	0.123	0.114	0.002	0.052	0.033	0.267	0.088	0.145
Ki67	EN	0.014	0.008	0.001	0.017	0.002	0.094	0.006	0.013
Ki67	LGBM	0.033	0.026	0.001	0.029	0.005	0.136	0.014	0.041
PR	AE	0.265	0.22	0.005	0.146	0.094	1.0	0.202	0.319
PR	EN	0.028	0.024	0.001	0.019	0.0	0.09	0.015	0.034
PR	LGBM	0.017	0.01	0.001	0.019	0.0	0.081	0.005	0.02
Vimentin	AE	0.141	0.144	0.002	0.052	0.046	0.259	0.092	0.183
Vimentin	EN	0.025	0.025	0.0	0.011	0.004	0.05	0.018	0.033
Vimentin	LGBM	0.033	0.03	0.0	0.014	0.008	0.084	0.022	0.04
aSMA	AE	0.538	0.52	0.005	0.172	0.253	0.964	0.404	0.658
aSMA	EN	0.09	0.085	0.001	0.039	0.012	0.185	0.059	0.113
aSMA	LGBM	0.11	0.095	0.002	0.067	0.022	0.292	0.056	0.158
p21	AE	0.145	0.135	0.002	0.059	0.056	0.318	0.11	0.156
p21	EN	0.032	0.026	0.001	0.02	0.006	0.08	0.018	0.042
p21	LGBM	0.019	0.012	0.001	0.02	0.004	0.092	0.009	0.019
pERK	AE	0.387	0.368	0.006	0.182	0.148	0.9	0.237	0.476
pERK	EN	0.049	0.046	0.001	0.021	0.012	0.102	0.034	0.059
pERK	LGBM	0.133	0.113	0.003	0.083	0.026	0.355	0.065	0.172
pRB	AE	0.189	0.178	0.003	0.1	0.025	0.45	0.119	0.261
pRB	EN	0.03	0.022	0.001	0.025	0.004	0.117	0.015	0.038
pRB	LGBM	0.019	0.014	0.001	0.017	0.004	0.087	0.01	0.019

Supplementary Table 11: Fig 5 TMA performance EN, LGBM & AE boxenplot description.

Marker	Model	Mean	Median	SEM	SD	Min	Max	Q1 (25%)	Q3 (75%)
AR	0 µm	0.088	0.072	0.0	0.055	0.049	0.227	0.059	0.083
AR	30 µm	0.08	0.069	0.0	0.04	0.042	0.185	0.047	0.082
AR	60 µm	0.07	0.071	0.0	0.022	0.046	0.112	0.049	0.084
CD45	0 µm	0.075	0.057	0.0	0.034	0.036	0.139	0.053	0.123
CD45	30 µm	0.08	0.077	0.0	0.034	0.036	0.187	0.051	0.123
CD45	60 µm	0.079	0.063	0.0	0.036	0.038	0.137	0.042	0.129
CK14	0 µm	0.108	0.127	0.0	0.05	0.032	0.193	0.059	0.156
CK14	30 µm	0.119	0.135	0.0	0.051	0.05	0.336	0.057	0.159
CK14	60 µm	0.105	0.134	0.0	0.051	0.034	0.17	0.049	0.15
CK17	0 µm	0.062	0.052	0.0	0.046	0.029	0.177	0.032	0.06
CK17	30 µm	0.064	0.056	0.0	0.045	0.03	0.181	0.038	0.058
CK17	60 µm	0.068	0.056	0.0	0.05	0.03	0.19	0.032	0.066
CK19	0 µm	0.211	0.229	0.001	0.088	0.02	0.308	0.201	0.296
CK19	30 µm	0.204	0.229	0.001	0.092	0.022	0.333	0.118	0.264
CK19	60 µm	0.206	0.234	0.001	0.095	0.019	0.324	0.139	0.273
EGFR	0 µm	0.073	0.066	0.0	0.042	0.027	0.161	0.04	0.105
EGFR	30 µm	0.064	0.04	0.0	0.037	0.031	0.186	0.032	0.07
EGFR	60 µm	0.065	0.04	0.0	0.046	0.028	0.173	0.035	0.07
ER	0 µm	0.151	0.139	0.001	0.075	0.054	0.589	0.117	0.217
ER	30 µm	0.158	0.133	0.001	0.08	0.059	0.613	0.064	0.238
ER	60 µm	0.129	0.13	0.001	0.056	0.061	0.343	0.069	0.144
Ecad	0 µm	0.117	0.124	0.0	0.045	0.038	0.181	0.087	0.166
Ecad	30 µm	0.111	0.095	0.0	0.042	0.039	0.182	0.085	0.138
Ecad	60 µm	0.108	0.096	0.0	0.043	0.037	0.18	0.082	0.139
HER2	0 µm	0.066	0.079	0.0	0.026	0.025	0.112	0.048	0.087

HER2	30 µm	0.069	0.074	0.0	0.028	0.035	0.266	0.044	0.096
HER2	60 µm	0.077	0.088	0.0	0.035	0.037	0.267	0.04	0.102
Ki67	0 µm	0.059	0.058	0.0	0.022	0.027	0.093	0.037	0.077
Ki67	30 µm	0.075	0.083	0.0	0.019	0.036	0.127	0.055	0.087
Ki67	60 µm	0.082	0.089	0.0	0.021	0.039	0.124	0.072	0.097
PR	0 µm	0.127	0.106	0.001	0.061	0.033	0.23	0.092	0.206
PR	30 µm	0.119	0.096	0.001	0.061	0.029	0.245	0.087	0.153
PR	60 µm	0.103	0.094	0.0	0.047	0.028	0.238	0.084	0.157
Vimentin	0 µm	0.12	0.101	0.0	0.039	0.057	0.185	0.092	0.158
Vimentin	30 µm	0.125	0.116	0.0	0.039	0.061	0.18	0.101	0.168
Vimentin	60 µm	0.127	0.115	0.0	0.04	0.059	0.172	0.1	0.168
aSMA	0 µm	0.085	0.094	0.0	0.031	0.023	0.12	0.067	0.113
aSMA	30 µm	0.09	0.1	0.0	0.031	0.024	0.139	0.071	0.108
aSMA	60 µm	0.094	0.102	0.0	0.036	0.024	0.142	0.069	0.12
p21	0 µm	0.085	0.073	0.0	0.037	0.025	0.145	0.065	0.125
p21	30 µm	0.096	0.089	0.0	0.041	0.025	0.168	0.059	0.126
p21	60 µm	0.086	0.062	0.0	0.038	0.046	0.159	0.056	0.125
pERK	0 µm	0.083	0.079	0.0	0.021	0.051	0.117	0.077	0.094
pERK	30 µm	0.088	0.075	0.0	0.031	0.054	0.326	0.065	0.115
pERK	60 µm	0.092	0.076	0.0	0.034	0.055	0.316	0.061	0.118
pRB	0 µm	0.08	0.077	0.0	0.015	0.053	0.113	0.076	0.085
pRB	30 µm	0.084	0.08	0.0	0.021	0.043	0.121	0.079	0.086
pRB	60 µm	0.08	0.084	0.0	0.017	0.043	0.104	0.069	0.098

Supplementary Table 12: Fig 6 LGBM Spatial Performance boxenplot description.

Marker	Model	Mean	Median	SEM	SD	Min	Max	Q1 (25%)	Q3 (75%)
AR	0 µm	0.13	0.107	0.002	0.057	0.014	0.464	0.094	0.138
AR	30 µm	0.096	0.099	0.001	0.043	0.013	0.597	0.059	0.13
AR	60 µm	0.108	0.098	0.001	0.054	0.012	0.597	0.067	0.16
CD45	0 µm	0.085	0.074	0.002	0.05	0.016	0.533	0.046	0.132
CD45	30 µm	0.1	0.085	0.001	0.051	0.016	0.275	0.06	0.126
CD45	60 µm	0.111	0.086	0.001	0.059	0.02	0.638	0.066	0.16
CK14	0 µm	0.13	0.133	0.002	0.05	0.017	0.432	0.076	0.161
CK14	30 µm	0.111	0.11	0.001	0.047	0.013	0.259	0.082	0.137
CK14	60 µm	0.107	0.116	0.001	0.052	0.013	0.23	0.066	0.148
CK17	0 µm	0.086	0.068	0.001	0.034	0.01	0.212	0.06	0.098
CK17	30 µm	0.075	0.068	0.001	0.034	0.022	0.307	0.06	0.075
CK17	60 µm	0.076	0.066	0.0	0.03	0.018	0.397	0.061	0.081
CK19	0 µm	0.239	0.249	0.003	0.106	0.025	0.826	0.203	0.294
CK19	30 µm	0.097	0.086	0.001	0.06	0.011	0.487	0.065	0.136
CK19	60 µm	0.117	0.118	0.001	0.05	0.016	0.356	0.093	0.155
EGFR	0 µm	0.079	0.074	0.002	0.063	0.013	0.59	0.036	0.126
EGFR	30 µm	0.073	0.062	0.001	0.039	0.012	0.342	0.051	0.088
EGFR	60 µm	0.087	0.074	0.001	0.057	0.014	0.59	0.065	0.096
ER	0 µm	0.177	0.162	0.002	0.071	0.02	0.654	0.124	0.21
ER	30 µm	0.125	0.112	0.001	0.062	0.011	0.722	0.074	0.176
ER	60 µm	0.13	0.113	0.001	0.067	0.011	0.527	0.077	0.194
Ecad	0 µm	0.146	0.144	0.002	0.075	0.019	0.47	0.107	0.185
Ecad	30 µm	0.134	0.11	0.001	0.083	0.013	0.47	0.101	0.143
Ecad	60 µm	0.157	0.137	0.002	0.1	0.014	0.434	0.129	0.148
HER2	0 µm	0.063	0.054	0.001	0.045	0.01	0.706	0.049	0.075

HER2	30 µm	0.1	0.086	0.001	0.049	0.01	0.306	0.066	0.118
HER2	60 µm	0.107	0.104	0.001	0.043	0.01	0.517	0.071	0.126
Ki67	0 µm	0.08	0.076	0.001	0.043	0.014	0.491	0.058	0.093
Ki67	30 µm	0.086	0.094	0.001	0.04	0.013	0.203	0.054	0.119
Ki67	60 µm	0.101	0.103	0.001	0.048	0.014	0.191	0.065	0.145
PR	0 µm	0.182	0.131	0.004	0.118	0.017	0.455	0.113	0.253
PR	30 µm	0.133	0.12	0.001	0.086	0.013	0.322	0.063	0.193
PR	60 µm	0.13	0.111	0.001	0.08	0.006	0.328	0.081	0.175
Vimentin	0 µm	0.171	0.165	0.002	0.052	0.025	0.505	0.143	0.199
Vimentin	30 µm	0.157	0.158	0.001	0.064	0.023	0.413	0.136	0.189
Vimentin	60 µm	0.164	0.166	0.001	0.063	0.023	0.505	0.14	0.205
aSMA	0 µm	0.105	0.111	0.001	0.038	0.022	0.327	0.095	0.129
aSMA	30 µm	0.097	0.11	0.001	0.035	0.013	0.248	0.084	0.121
aSMA	60 µm	0.104	0.115	0.001	0.037	0.013	0.438	0.098	0.127
p21	0 µm	0.122	0.092	0.004	0.125	0.016	0.487	0.07	0.104
p21	30 µm	0.09	0.1	0.001	0.04	0.014	0.487	0.056	0.121
p21	60 µm	0.096	0.109	0.001	0.039	0.013	0.487	0.054	0.129
pERK	0 µm	0.103	0.112	0.002	0.053	0.017	0.65	0.072	0.129
pERK	30 µm	0.1	0.098	0.001	0.06	0.014	0.65	0.065	0.133
pERK	60 µm	0.109	0.108	0.001	0.067	0.015	0.389	0.064	0.138
pRB	0 µm	0.155	0.102	0.004	0.138	0.015	0.549	0.092	0.144
pRB	30 µm	0.109	0.098	0.001	0.045	0.011	0.548	0.087	0.146
pRB	60 µm	0.112	0.107	0.001	0.054	0.013	0.286	0.083	0.122

Supplementary Table 13: Fig 7a AE Spatial Performance boxenplot description

Marker	Model	Mean	Median	SEM	SD	Min	Max	Q1 (25%)	Q3 (75%)
AR	0 µm	0.124	0.101	0.002	0.07	0.015	0.291	0.08	0.17
AR	30 µm	0.096	0.076	0.001	0.056	0.018	0.258	0.052	0.12
AR	60 µm	0.106	0.093	0.001	0.056	0.018	0.248	0.064	0.141
CD45	0 µm	0.099	0.073	0.001	0.055	0.017	0.318	0.06	0.139
CD45	30 µm	0.095	0.08	0.001	0.048	0.019	0.244	0.059	0.13
CD45	60 µm	0.098	0.078	0.001	0.056	0.019	0.267	0.063	0.117
CK14	0 µm	0.132	0.133	0.001	0.055	0.019	0.265	0.09	0.185
CK14	30 µm	0.113	0.089	0.002	0.075	0.023	0.269	0.046	0.191
CK14	60 µm	0.116	0.104	0.002	0.072	0.024	0.24	0.05	0.17
CK17	0 µm	0.084	0.068	0.001	0.039	0.008	0.212	0.063	0.095
CK17	30 µm	0.083	0.069	0.001	0.042	0.008	0.197	0.062	0.084
CK17	60 µm	0.078	0.068	0.001	0.034	0.007	0.212	0.062	0.077
CK19	0 µm	0.203	0.209	0.002	0.077	0.029	0.303	0.141	0.274
CK19	30 µm	0.189	0.198	0.002	0.075	0.031	0.29	0.13	0.259
CK19	60 µm	0.18	0.199	0.002	0.074	0.046	0.306	0.098	0.24
EGFR	0 µm	0.094	0.085	0.001	0.054	0.016	0.292	0.049	0.157
EGFR	30 µm	0.086	0.082	0.001	0.046	0.019	0.21	0.052	0.114
EGFR	60 µm	0.077	0.072	0.001	0.035	0.017	0.239	0.045	0.101
ER	0 µm	0.167	0.157	0.001	0.055	0.019	0.282	0.138	0.211
ER	30 µm	0.136	0.157	0.001	0.064	0.015	0.328	0.103	0.188
ER	60 µm	0.144	0.148	0.002	0.074	0.016	0.316	0.102	0.185
Ecad	0 µm	0.143	0.146	0.001	0.064	0.017	0.3	0.121	0.16
Ecad	30 µm	0.143	0.132	0.002	0.073	0.016	0.332	0.121	0.151
Ecad	60 µm	0.149	0.147	0.002	0.067	0.017	0.334	0.132	0.167
HER2	0 µm	0.068	0.058	0.001	0.041	0.009	0.325	0.047	0.075

HER2	30 µm	0.084	0.077	0.001	0.035	0.01	0.197	0.063	0.115
HER2	60 µm	0.088	0.08	0.001	0.041	0.009	0.306	0.063	0.118
Ki67	0 µm	0.074	0.082	0.001	0.031	0.014	0.281	0.056	0.097
Ki67	30 µm	0.085	0.097	0.001	0.035	0.013	0.281	0.059	0.11
Ki67	60 µm	0.107	0.111	0.001	0.059	0.014	0.282	0.07	0.133
PR	0 µm	0.133	0.13	0.001	0.06	0.017	0.318	0.089	0.193
PR	30 µm	0.106	0.099	0.001	0.042	0.017	0.318	0.083	0.136
PR	60 µm	0.112	0.115	0.001	0.049	0.018	0.318	0.085	0.152
Vimentin	0 µm	0.14	0.137	0.001	0.051	0.039	0.33	0.111	0.181
Vimentin	30 µm	0.159	0.171	0.001	0.045	0.053	0.281	0.127	0.186
Vimentin	60 µm	0.174	0.193	0.001	0.058	0.056	0.277	0.154	0.209
aSMA	0 µm	0.127	0.129	0.001	0.044	0.029	0.258	0.115	0.149
aSMA	30 µm	0.112	0.124	0.001	0.036	0.03	0.222	0.09	0.14
aSMA	60 µm	0.128	0.135	0.001	0.043	0.032	0.273	0.111	0.16
p21	0 µm	0.072	0.069	0.001	0.028	0.016	0.247	0.054	0.097
p21	30 µm	0.086	0.097	0.001	0.038	0.019	0.159	0.055	0.113
p21	60 µm	0.09	0.104	0.001	0.036	0.016	0.186	0.056	0.124
pERK	0 µm	0.101	0.109	0.001	0.047	0.017	0.321	0.067	0.141
pERK	30 µm	0.101	0.095	0.001	0.049	0.018	0.257	0.071	0.125
pERK	60 µm	0.101	0.098	0.001	0.053	0.017	0.228	0.062	0.126
pRB	0 µm	0.111	0.115	0.001	0.054	0.016	0.283	0.069	0.157
pRB	30 µm	0.105	0.102	0.001	0.029	0.018	0.165	0.085	0.126
pRB	60 µm	0.093	0.082	0.001	0.04	0.016	0.172	0.07	0.118

Supplementary Table 14: Fig 7b AE M Spatial Performance boxenplot description

Marker	Model	Mean	Median	SEM	SD	Min	Max	Q1 (25%)	Q3 (75%)
AE	0 µm	0.128	0.112	0.001	0.09	0.01	0.826	0.069	0.161
AE	30 µm	0.105	0.099	0.0	0.059	0.01	0.722	0.065	0.134
AE	60 µm	0.113	0.109	0.0	0.063	0.006	0.638	0.068	0.142
AE M	0 µm	0.115	0.109	0.0	0.062	0.008	0.33	0.066	0.154
AE M	30 µm	0.111	0.102	0.0	0.059	0.008	0.332	0.065	0.144
AE M	60 µm	0.115	0.105	0.0	0.062	0.007	0.334	0.066	0.152
LGBM	0 µm	0.099	0.084	0.0	0.061	0.02	0.589	0.056	0.124
LGBM	30 µm	0.102	0.086	0.0	0.06	0.022	0.613	0.059	0.127
LGBM	60 µm	0.098	0.087	0.0	0.056	0.019	0.343	0.057	0.128

Supplementary Table 15: Fig 7c Performance comparison between LGBM, AE & AE M models

Marker	Model	Mean	Median	SEM	SD	Min	Max	Q1 (25%)	Q3 (75%)
AR	Ground Truth Data	0.697	0.712	0.003	0.177	0.453	0.947	0.52	0.825
AR	Imputed Data	0.89	0.91	0.002	0.091	0.712	0.997	0.879	0.938
AR	Removed Data	0.753	0.754	0.002	0.127	0.531	0.948	0.64	0.818
CD45	Ground Truth Data	0.697	0.72	0.003	0.177	0.455	0.946	0.523	0.822
CD45	Imputed Data	0.715	0.71	0.003	0.16	0.462	0.944	0.565	0.824
CD45	Removed Data	0.686	0.697	0.003	0.185	0.45	0.944	0.507	0.824
CK14	Ground Truth Data	0.702	0.709	0.003	0.173	0.454	0.947	0.531	0.826
CK14	Imputed Data	0.807	0.793	0.001	0.071	0.706	0.908	0.755	0.873
CK14	Removed Data	0.694	0.72	0.003	0.187	0.42	0.946	0.49	0.826
CK17	Ground Truth Data	0.701	0.718	0.003	0.176	0.455	0.946	0.519	0.825
CK17	Imputed Data	0.881	0.901	0.001	0.084	0.736	1.0	0.798	0.931
CK17	Removed Data	0.698	0.714	0.003	0.176	0.454	0.946	0.521	0.822
CK19	Ground Truth Data	0.696	0.711	0.003	0.178	0.464	0.945	0.517	0.825
CK19	Imputed Data	0.843	0.882	0.001	0.078	0.72	0.99	0.78	0.918
CK19	Removed Data	0.681	0.716	0.003	0.189	0.379	0.937	0.533	0.792
EGFR	Ground Truth Data	0.7	0.716	0.003	0.177	0.439	0.946	0.522	0.822
EGFR	Imputed Data	0.766	0.768	0.002	0.094	0.58	0.91	0.707	0.815
EGFR	Removed Data	0.708	0.718	0.003	0.168	0.468	0.944	0.54	0.821

ER	Ground Truth Data	0.702	0.714	0.003	0.174	0.466	0.945	0.525	0.824
ER	Imputed Data	0.857	0.897	0.002	0.106	0.697	1.0	0.76	0.931
ER	Removed Data	0.567	0.616	0.005	0.307	0.125	0.947	0.347	0.804
Ecad	Ground Truth Data	0.697	0.704	0.003	0.176	0.455	0.946	0.529	0.821
Ecad	Imputed Data	0.736	0.766	0.002	0.131	0.487	0.91	0.616	0.819
Ecad	Removed Data	0.705	0.716	0.003	0.174	0.445	0.948	0.526	0.825
HER2	Ground Truth Data	0.698	0.717	0.003	0.177	0.464	0.945	0.511	0.824
HER2	Imputed Data	0.745	0.769	0.002	0.121	0.525	0.91	0.703	0.851
HER2	Removed Data	0.692	0.703	0.003	0.182	0.422	0.946	0.518	0.822
Ki67	Ground Truth Data	0.696	0.707	0.003	0.179	0.443	0.946	0.517	0.823
Ki67	Imputed Data	0.751	0.747	0.002	0.112	0.532	0.91	0.707	0.814
Ki67	Removed Data	0.701	0.709	0.003	0.173	0.465	0.945	0.526	0.824
PR	Ground Truth Data	0.698	0.718	0.003	0.176	0.439	0.946	0.524	0.824
PR	Imputed Data	0.866	0.868	0.001	0.069	0.769	0.965	0.8	0.918
PR	Removed Data	0.741	0.773	0.003	0.163	0.431	0.945	0.665	0.825
Vimentin	Ground Truth Data	0.7	0.712	0.003	0.175	0.466	0.946	0.517	0.822
Vimentin	Imputed Data	0.737	0.726	0.002	0.119	0.538	0.908	0.628	0.819
Vimentin	Removed Data	0.701	0.703	0.003	0.173	0.42	0.946	0.544	0.824
aSMA	Ground Truth Data	0.698	0.713	0.003	0.177	0.441	0.946	0.518	0.822

aSMA	Imputed Data	0.815	0.851	0.002	0.114	0.608	0.934	0.75	0.912
aSMA	Removed Data	0.707	0.729	0.003	0.173	0.451	0.945	0.528	0.827
p21	Ground Truth Data	0.699	0.715	0.003	0.177	0.456	0.945	0.523	0.82
p21	Imputed Data	0.71	0.722	0.003	0.151	0.418	0.91	0.621	0.811
p21	Removed Data	0.695	0.716	0.003	0.181	0.447	0.947	0.508	0.823
pERK	Ground Truth Data	0.699	0.727	0.003	0.176	0.456	0.946	0.513	0.824
pERK	Imputed Data	0.676	0.712	0.003	0.199	0.292	0.91	0.573	0.818
pERK	Removed Data	0.69	0.723	0.003	0.192	0.327	0.947	0.576	0.822
pRB	Ground Truth Data	0.699	0.719	0.003	0.176	0.459	0.947	0.519	0.822
pRB	Imputed Data	0.814	0.81	0.001	0.071	0.71	0.91	0.78	0.873
pRB	Removed Data	0.714	0.713	0.003	0.163	0.454	0.947	0.587	0.824

Supplementary Table 16: Fig 8 Downstream Accuracy for Ground truth removed and imputed data boxen plot description

Marker	Model	Mean	Median	SEM	SD	Min	Max	Q1 (25%)	Q3 (75%)
AR	AP	0.093	0.087	0.003	0.055	0.016	0.216	0.063	0.109
AR	IP	0.118	0.112	0.003	0.053	0.043	0.205	0.075	0.145
CD45	AP	0.109	0.108	0.003	0.042	0.049	0.178	0.069	0.155
CD45	IP	0.116	0.094	0.004	0.067	0.045	0.27	0.061	0.153
CK14	AP	0.119	0.118	0.003	0.047	0.058	0.242	0.077	0.15
CK14	IP	0.064	0.045	0.003	0.046	0.017	0.166	0.022	0.094
CK17	AP	0.066	0.058	0.002	0.032	0.028	0.145	0.05	0.071
CK17	IP	0.087	0.061	0.004	0.06	0.031	0.24	0.055	0.079
CK19	AP	0.256	0.289	0.005	0.082	0.086	0.399	0.214	0.318
CK19	IP	0.145	0.146	0.003	0.047	0.068	0.227	0.117	0.159
EGFR	AP	0.079	0.058	0.003	0.047	0.03	0.185	0.039	0.117
EGFR	IP	0.118	0.121	0.002	0.03	0.053	0.173	0.106	0.131
ER	AP	0.192	0.165	0.004	0.061	0.087	0.332	0.141	0.255
ER	IP	0.104	0.091	0.002	0.026	0.06	0.164	0.085	0.117
Ecad	AP	0.112	0.113	0.003	0.041	0.019	0.196	0.104	0.123
Ecad	IP	0.126	0.102	0.003	0.051	0.061	0.236	0.084	0.156
HER2	AP	0.08	0.07	0.002	0.033	0.038	0.138	0.057	0.117
HER2	IP	0.059	0.052	0.002	0.026	0.017	0.11	0.044	0.07
Ki67	AP	0.069	0.065	0.002	0.024	0.021	0.114	0.055	0.079
Ki67	IP	0.088	0.073	0.003	0.046	0.025	0.144	0.041	0.138
Mean	AP	0.112	0.111	0.009	0.025	0.073	0.147	0.097	0.133
Mean	IP	0.105	0.099	0.007	0.019	0.085	0.138	0.091	0.116
PR	AP	0.137	0.125	0.004	0.063	0.03	0.273	0.101	0.171
PR	IP	0.092	0.107	0.004	0.063	0.005	0.196	0.03	0.122
Vimentin	AP	0.137	0.128	0.003	0.051	0.049	0.268	0.106	0.149
Vimentin	IP	0.125	0.118	0.003	0.049	0.058	0.221	0.086	0.161
aSMA	AP	0.098	0.106	0.002	0.029	0.033	0.139	0.083	0.115
aSMA	IP	0.111	0.119	0.002	0.024	0.05	0.154	0.096	0.126
p21	AP	0.086	0.078	0.003	0.042	0.022	0.171	0.052	0.11
p21	IP	0.09	0.055	0.004	0.064	0.037	0.245	0.048	0.119
pERK	AP	0.077	0.08	0.002	0.027	0.025	0.115	0.057	0.102
pERK	IP	0.12	0.116	0.004	0.061	0.051	0.267	0.076	0.133
pRB	AP	0.086	0.085	0.002	0.039	0.015	0.153	0.06	0.123
pRB	IP	0.114	0.115	0.003	0.041	0.066	0.182	0.077	0.152

Supplementary Table 17: IP vs AP performance for Elastic Net boxenplot description.

Marker	Model	Mean	Median	SEM	SD	Min	Max	Q1 (25%)	Q3 (75%)
AR	AP	0.088	0.072	0.0	0.055	0.049	0.227	0.059	0.083
AR	IP	0.1	0.088	0.0	0.051	0.04	0.193	0.059	0.133
CD45	AP	0.075	0.057	0.0	0.034	0.036	0.139	0.053	0.123
CD45	IP	0.103	0.091	0.0	0.051	0.037	0.169	0.059	0.157
CK14	AP	0.108	0.127	0.0	0.05	0.032	0.193	0.059	0.156
CK14	IP	0.056	0.034	0.0	0.043	0.018	0.151	0.021	0.085
CK17	AP	0.062	0.052	0.0	0.046	0.029	0.177	0.032	0.06
CK17	IP	0.082	0.06	0.001	0.062	0.037	0.25	0.047	0.078
CK19	AP	0.211	0.229	0.001	0.088	0.02	0.308	0.201	0.296
CK19	IP	0.128	0.121	0.0	0.046	0.068	0.202	0.086	0.169
EGFR	AP	0.073	0.066	0.0	0.042	0.027	0.161	0.04	0.105
EGFR	IP	0.113	0.12	0.0	0.041	0.049	0.18	0.088	0.138
ER	AP	0.151	0.139	0.001	0.075	0.054	0.589	0.117	0.217
ER	IP	0.105	0.108	0.0	0.018	0.07	0.133	0.092	0.117
Ecad	AP	0.117	0.124	0.0	0.045	0.038	0.181	0.087	0.166
Ecad	IP	0.08	0.077	0.0	0.031	0.029	0.142	0.063	0.098
HER2	AP	0.066	0.079	0.0	0.026	0.025	0.112	0.048	0.087
HER2	IP	0.07	0.052	0.0	0.045	0.033	0.182	0.043	0.067
Ki67	AP	0.059	0.058	0.0	0.022	0.027	0.093	0.037	0.077
Ki67	IP	0.077	0.083	0.0	0.038	0.025	0.121	0.041	0.112
Mean	AP	0.098	0.1	0.009	0.026	0.053	0.128	0.085	0.116
Mean	IP	0.092	0.082	0.007	0.021	0.075	0.136	0.08	0.103
PR	AP	0.127	0.106	0.001	0.061	0.033	0.23	0.092	0.206
PR	IP	0.08	0.089	0.0	0.047	0.005	0.152	0.079	0.121
Vimentin	AP	0.12	0.101	0.0	0.039	0.057	0.185	0.092	0.158
Vimentin	IP	0.13	0.111	0.0	0.042	0.085	0.22	0.102	0.152
aSMA	AP	0.085	0.094	0.0	0.031	0.023	0.12	0.067	0.113
aSMA	IP	0.088	0.084	0.0	0.034	0.041	0.157	0.06	0.108
p21	AP	0.085	0.073	0.0	0.037	0.025	0.145	0.065	0.125
p21	IP	0.07	0.059	0.0	0.031	0.03	0.127	0.049	0.086
pERK	AP	0.083	0.079	0.0	0.021	0.051	0.117	0.077	0.094
pERK	IP	0.116	0.1	0.001	0.061	0.064	0.269	0.075	0.122
pRB	AP	0.08	0.077	0.0	0.015	0.053	0.113	0.076	0.085
pRB	IP	0.08	0.076	0.0	0.024	0.044	0.139	0.071	0.083

Supplementary Table 18: IP vs AP performance for LGBM boxen plot descriptions

Marker	Model	Mean	Median	SEM	SD	Min	Max	Q1 (25%)	Q3 (75%)
AR	AP	0.129	0.107	0.002	0.055	0.014	0.262	0.094	0.138
AR	IP	0.125	0.139	0.0	0.053	0.016	0.223	0.076	0.164
CD45	AP	0.083	0.073	0.001	0.045	0.016	0.227	0.046	0.132
CD45	IP	0.123	0.092	0.0	0.054	0.052	0.318	0.083	0.154
CK14	AP	0.13	0.133	0.002	0.049	0.017	0.253	0.076	0.161
CK14	IP	0.058	0.033	0.0	0.046	0.01	0.207	0.031	0.067
CK17	AP	0.086	0.068	0.001	0.034	0.01	0.212	0.06	0.098
CK17	IP	0.089	0.061	0.0	0.065	0.027	0.307	0.049	0.086
CK19	AP	0.214	0.234	0.003	0.083	0.025	0.336	0.202	0.261
CK19	IP	0.177	0.195	0.0	0.06	0.021	0.314	0.103	0.228
EGFR	AP	0.077	0.066	0.002	0.056	0.013	0.239	0.036	0.125
EGFR	IP	0.114	0.114	0.0	0.045	0.025	0.302	0.081	0.132
ER	AP	0.172	0.152	0.002	0.057	0.02	0.328	0.124	0.209
ER	IP	0.115	0.12	0.0	0.027	0.016	0.2	0.097	0.136
Ecad	AP	0.145	0.143	0.002	0.073	0.019	0.313	0.107	0.182
Ecad	IP	0.119	0.108	0.0	0.051	0.015	0.317	0.09	0.121
HER2	AP	0.062	0.054	0.001	0.036	0.01	0.325	0.049	0.075
HER2	IP	0.078	0.068	0.0	0.037	0.017	0.306	0.054	0.081
Ki67	AP	0.079	0.076	0.001	0.04	0.014	0.208	0.058	0.093
Ki67	IP	0.081	0.075	0.0	0.039	0.015	0.281	0.046	0.124
Mean	AP	0.118	0.123	0.012	0.033	0.055	0.161	0.1	0.134
Mean	IP	0.106	0.097	0.008	0.023	0.082	0.157	0.096	0.109
PR	AP	0.151	0.129	0.003	0.079	0.017	0.318	0.113	0.207
PR	IP	0.088	0.103	0.0	0.063	0.009	0.318	0.017	0.12
Vimentin	AP	0.169	0.165	0.001	0.047	0.025	0.313	0.142	0.194
Vimentin	IP	0.15	0.125	0.0	0.055	0.03	0.32	0.107	0.197
aSMA	AP	0.105	0.111	0.001	0.038	0.022	0.327	0.095	0.129
aSMA	IP	0.129	0.133	0.0	0.044	0.013	0.327	0.094	0.154
p21	AP	0.082	0.092	0.001	0.031	0.016	0.198	0.07	0.103
p21	IP	0.091	0.063	0.0	0.044	0.029	0.292	0.056	0.137
pERK	AP	0.101	0.11	0.001	0.042	0.017	0.321	0.072	0.128
pERK	IP	0.112	0.104	0.0	0.046	0.065	0.305	0.078	0.124
pRB	AP	0.11	0.1	0.001	0.045	0.015	0.246	0.091	0.142
pRB	IP	0.132	0.109	0.0	0.064	0.011	0.338	0.089	0.158

Supplementary Table 19: IP vs AP performance for AE boxen plot descriptions

Marker	Model	Mean	Median	SEM	SD	Min	Max	Q1 (25%)	Q3 (75%)
CD45	Imputed Silhouette Score	0.263	0.194	0.003	0.222	0.112	0.84	0.139	0.238
CD45	Original Silhouette Score	0.278	0.215	0.003	0.232	0.089	0.87	0.162	0.25
CK14	Imputed Silhouette Score	0.256	0.193	0.003	0.237	0.076	0.874	0.145	0.218
CK14	Original Silhouette Score	0.278	0.215	0.003	0.232	0.089	0.87	0.162	0.25
CK17	Imputed Silhouette Score	0.247	0.171	0.003	0.247	0.025	0.864	0.107	0.275
CK17	Original Silhouette Score	0.278	0.215	0.003	0.232	0.089	0.87	0.162	0.25
CK19	Imputed Silhouette Score	0.2	0.122	0.003	0.254	-0.008	0.85	0.065	0.181
CK19	Original Silhouette Score	0.278	0.215	0.003	0.232	0.089	0.87	0.162	0.25
aSMA	Imputed Silhouette Score	0.275	0.193	0.003	0.23	0.119	0.867	0.141	0.261
aSMA	Original Silhouette Score	0.278	0.215	0.003	0.232	0.089	0.87	0.162	0.25

Supplementary Table 20: Silhouette Scores for phenotype calling between Original & Imputed data boxen plot descriptions.

Marker	Mean	Median	SEM	SD	Min	Max	Q1 (25%)	Q3 (75%)
CD45	0.718	0.709	0.008	0.117	0.517	0.876	0.66	0.811
CK14	0.815	0.85	0.006	0.093	0.625	0.948	0.766	0.869
CK17	0.8	0.862	0.009	0.145	0.535	0.944	0.744	0.902
CK19	0.632	0.644	0.01	0.157	0.35	0.925	0.559	0.708
aSMA	0.666	0.639	0.007	0.115	0.485	0.903	0.599	0.725

Supplementary Table 21: AMI Scores for phenotype calling for specific markers boxen plot descriptions.

Marker	Model	Mean	Median	SEM	SD	Min	Max	Q1 (25%)	Q3 (75%)
AR	0 µm	0.088	0.072	0.0	0.055	0.049	0.227	0.059	0.083
AR	120 µm	0.088	0.082	0.0	0.04	0.045	0.183	0.055	0.103
AR	15 µm	0.085	0.076	0.0	0.046	0.041	0.2	0.058	0.093
AR	30 µm	0.08	0.069	0.0	0.04	0.042	0.185	0.047	0.082
AR	60 µm	0.07	0.071	0.0	0.022	0.046	0.112	0.049	0.084
AR	90 µm	0.088	0.083	0.0	0.045	0.043	0.188	0.049	0.111
CD45	0 µm	0.075	0.057	0.0	0.034	0.036	0.139	0.053	0.123
CD45	120 µm	0.077	0.063	0.0	0.033	0.04	0.141	0.051	0.09
CD45	15 µm	0.075	0.063	0.0	0.033	0.036	0.148	0.056	0.125
CD45	30 µm	0.08	0.077	0.0	0.034	0.036	0.187	0.051	0.123
CD45	60 µm	0.079	0.063	0.0	0.036	0.038	0.137	0.042	0.129
CD45	90 µm	0.074	0.057	0.0	0.034	0.039	0.163	0.043	0.09
CK14	0 µm	0.108	0.127	0.0	0.05	0.032	0.193	0.059	0.156
CK14	120 µm	0.106	0.13	0.0	0.051	0.028	0.18	0.054	0.147
CK14	15 µm	0.122	0.144	0.0	0.052	0.046	0.275	0.063	0.174
CK14	30 µm	0.119	0.135	0.0	0.051	0.05	0.336	0.057	0.159
CK14	60 µm	0.105	0.134	0.0	0.051	0.034	0.17	0.049	0.15
CK14	90 µm	0.11	0.139	0.0	0.047	0.035	0.163	0.056	0.149
CK17	0 µm	0.062	0.052	0.0	0.046	0.029	0.177	0.032	0.06
CK17	120 µm	0.064	0.058	0.0	0.042	0.027	0.174	0.041	0.072
CK17	15 µm	0.059	0.051	0.0	0.041	0.031	0.167	0.035	0.058
CK17	30 µm	0.064	0.056	0.0	0.045	0.03	0.181	0.038	0.058
CK17	60 µm	0.068	0.056	0.0	0.05	0.03	0.19	0.032	0.066
CK17	90 µm	0.066	0.05	0.0	0.047	0.026	0.179	0.041	0.061

CK19	0 µm	0.211	0.229	0.0	0.088	0.02	0.308	0.201	0.296
CK19	120 µm	0.229	0.257	0.001	0.099	0.022	0.348	0.148	0.264
CK19	15 µm	0.206	0.226	0.001	0.08	0.022	0.323	0.182	0.264
CK19	30 µm	0.204	0.229	0.001	0.092	0.022	0.333	0.118	0.264
CK19	60 µm	0.206	0.234	0.001	0.095	0.019	0.324	0.139	0.273
CK19	90 µm	0.243	0.26	0.001	0.096	0.022	0.364	0.216	0.27
EGFR	0 µm	0.073	0.066	0.0	0.042	0.027	0.161	0.04	0.105
EGFR	120 µm	0.071	0.067	0.0	0.046	0.029	0.177	0.036	0.083
EGFR	15 µm	0.068	0.065	0.0	0.039	0.029	0.253	0.039	0.103
EGFR	30 µm	0.064	0.04	0.0	0.037	0.031	0.186	0.032	0.07
EGFR	60 µm	0.065	0.04	0.0	0.046	0.028	0.173	0.035	0.07
EGFR	90 µm	0.072	0.067	0.0	0.047	0.026	0.17	0.032	0.108
ER	0 µm	0.151	0.139	0.0	0.075	0.054	0.589	0.117	0.217
ER	120 µm	0.194	0.155	0.001	0.111	0.057	0.431	0.112	0.337
ER	15 µm	0.153	0.137	0.001	0.076	0.056	0.303	0.109	0.217
ER	30 µm	0.158	0.133	0.001	0.08	0.059	0.613	0.064	0.238
ER	60 µm	0.129	0.13	0.001	0.056	0.061	0.343	0.069	0.144
ER	90 µm	0.152	0.149	0.001	0.064	0.056	0.351	0.133	0.183
Ecad	0 µm	0.117	0.124	0.0	0.045	0.038	0.181	0.087	0.166
Ecad	120 µm	0.117	0.102	0.0	0.045	0.038	0.353	0.1	0.138
Ecad	15 µm	0.114	0.1	0.0	0.04	0.039	0.179	0.086	0.153
Ecad	30 µm	0.111	0.095	0.0	0.042	0.039	0.182	0.085	0.138
Ecad	60 µm	0.108	0.096	0.0	0.043	0.037	0.18	0.082	0.139
Ecad	90 µm	0.119	0.124	0.0	0.041	0.037	0.179	0.099	0.136
HER2	0 µm	0.066	0.079	0.0	0.026	0.025	0.112	0.048	0.087

HER2	120 µm	0.087	0.103	0.0	0.036	0.041	0.185	0.044	0.107
HER2	15 µm	0.068	0.086	0.0	0.024	0.03	0.12	0.044	0.09
HER2	30 µm	0.069	0.074	0.0	0.028	0.035	0.266	0.044	0.096
HER2	60 µm	0.077	0.088	0.0	0.035	0.037	0.267	0.04	0.102
HER2	90 µm	0.079	0.095	0.0	0.031	0.037	0.171	0.051	0.1
Ki67	0 µm	0.059	0.058	0.0	0.022	0.027	0.093	0.037	0.077
Ki67	120 µm	0.069	0.073	0.0	0.02	0.03	0.093	0.063	0.091
Ki67	15 µm	0.064	0.055	0.0	0.023	0.038	0.138	0.039	0.081
Ki67	30 µm	0.075	0.083	0.0	0.019	0.036	0.127	0.055	0.087
Ki67	60 µm	0.082	0.089	0.0	0.021	0.039	0.124	0.072	0.097
Ki67	90 µm	0.078	0.08	0.0	0.015	0.041	0.101	0.075	0.088
PR	0 µm	0.127	0.106	0.0	0.061	0.033	0.23	0.092	0.206
PR	120 µm	0.118	0.095	0.001	0.06	0.024	0.361	0.093	0.16
PR	15 µm	0.128	0.102	0.001	0.059	0.034	0.238	0.092	0.188
PR	30 µm	0.119	0.096	0.001	0.061	0.029	0.245	0.087	0.153
PR	60 µm	0.103	0.094	0.0	0.047	0.028	0.238	0.084	0.157
PR	90 µm	0.121	0.096	0.001	0.057	0.025	0.216	0.09	0.178
Vimentin	0 µm	0.12	0.101	0.0	0.039	0.057	0.185	0.092	0.158
Vimentin	120 µm	0.12	0.109	0.0	0.04	0.054	0.205	0.105	0.167
Vimentin	15 µm	0.126	0.124	0.0	0.038	0.061	0.184	0.097	0.162
Vimentin	30 µm	0.125	0.116	0.0	0.039	0.061	0.18	0.101	0.168
Vimentin	60 µm	0.127	0.115	0.0	0.04	0.059	0.172	0.1	0.168
Vimentin	90 µm	0.123	0.113	0.0	0.037	0.061	0.206	0.108	0.154
aSMA	0 µm	0.085	0.094	0.0	0.031	0.023	0.12	0.067	0.113
aSMA	120 µm	0.092	0.102	0.0	0.035	0.025	0.157	0.077	0.108

aSMA	15 µm	0.089	0.101	0.0	0.03	0.023	0.138	0.069	0.11
aSMA	30 µm	0.09	0.1	0.0	0.031	0.024	0.139	0.071	0.108
aSMA	60 µm	0.094	0.102	0.0	0.036	0.024	0.142	0.069	0.12
aSMA	90 µm	0.086	0.082	0.0	0.03	0.02	0.138	0.066	0.109
p21	0 µm	0.085	0.073	0.0	0.037	0.025	0.145	0.065	0.125
p21	120 µm	0.101	0.099	0.0	0.043	0.024	0.195	0.06	0.116
p21	15 µm	0.091	0.089	0.0	0.041	0.026	0.163	0.058	0.125
p21	30 µm	0.096	0.089	0.0	0.041	0.025	0.168	0.059	0.126
p21	60 µm	0.086	0.062	0.0	0.038	0.046	0.159	0.056	0.125
p21	90 µm	0.092	0.088	0.0	0.038	0.025	0.162	0.056	0.124
pERK	0 µm	0.083	0.079	0.0	0.021	0.051	0.117	0.077	0.094
pERK	120 µm	0.092	0.083	0.0	0.028	0.048	0.168	0.069	0.118
pERK	15 µm	0.086	0.077	0.0	0.027	0.045	0.207	0.067	0.113
pERK	30 µm	0.088	0.075	0.0	0.031	0.054	0.326	0.065	0.115
pERK	60 µm	0.092	0.076	0.0	0.034	0.055	0.316	0.061	0.118
pERK	90 µm	0.087	0.073	0.0	0.031	0.054	0.316	0.067	0.116
pRB	0 µm	0.08	0.077	0.0	0.015	0.053	0.113	0.076	0.085
pRB	120 µm	0.084	0.083	0.0	0.018	0.049	0.111	0.076	0.104
pRB	15 µm	0.081	0.08	0.0	0.017	0.05	0.114	0.077	0.091
pRB	30 µm	0.084	0.08	0.0	0.021	0.043	0.121	0.079	0.086
pRB	60 µm	0.08	0.084	0.0	0.017	0.043	0.104	0.069	0.098
pRB	90 µm	0.084	0.085	0.0	0.018	0.047	0.211	0.072	0.101

Supplementary Table 22: Supplementary Figure 7 Spatial performance of LGBM

Marker	Model	Mean	Median	SEM	SD	Min	Max	Q1 (25%)	Q3 (75%)
AR	0 µm	0.13	0.107	0.001	0.057	0.014	0.464	0.094	0.138
AR	120 µm	0.112	0.114	0.001	0.061	0.013	0.464	0.053	0.169
AR	15 µm	0.096	0.1	0.001	0.041	0.011	0.597	0.061	0.129
AR	30 µm	0.096	0.099	0.001	0.043	0.013	0.597	0.059	0.13
AR	60 µm	0.108	0.098	0.001	0.054	0.012	0.597	0.067	0.16
AR	90 µm	0.112	0.104	0.001	0.067	0.014	0.464	0.057	0.161
CD45	0 µm	0.085	0.074	0.001	0.05	0.016	0.533	0.046	0.132
CD45	120 µm	0.106	0.079	0.001	0.064	0.018	0.286	0.063	0.116
CD45	15 µm	0.096	0.075	0.001	0.065	0.016	0.394	0.059	0.137
CD45	30 µm	0.1	0.085	0.001	0.051	0.016	0.275	0.06	0.126
CD45	60 µm	0.111	0.086	0.001	0.059	0.02	0.638	0.066	0.16
CD45	90 µm	0.115	0.084	0.001	0.07	0.042	0.318	0.061	0.161
CK14	0 µm	0.13	0.133	0.001	0.05	0.017	0.432	0.076	0.161
CK14	120 µm	0.108	0.112	0.001	0.049	0.013	0.692	0.069	0.132
CK14	15 µm	0.127	0.124	0.001	0.061	0.011	0.276	0.09	0.155
CK14	30 µm	0.111	0.11	0.001	0.047	0.013	0.259	0.082	0.137
CK14	60 µm	0.107	0.116	0.001	0.052	0.013	0.23	0.066	0.148
CK14	90 µm	0.104	0.11	0.001	0.05	0.013	0.692	0.063	0.132
CK17	0 µm	0.086	0.068	0.001	0.034	0.01	0.212	0.06	0.098
CK17	120 µm	0.076	0.068	0.0	0.025	0.034	0.307	0.062	0.081
CK17	15 µm	0.079	0.067	0.001	0.04	0.01	0.307	0.062	0.077
CK17	30 µm	0.075	0.068	0.001	0.034	0.022	0.307	0.06	0.075
CK17	60 µm	0.076	0.066	0.0	0.03	0.018	0.397	0.061	0.081
CK17	90 µm	0.079	0.068	0.0	0.032	0.01	0.397	0.06	0.103

CK19	0 µm	0.239	0.249	0.002	0.106	0.025	0.826	0.203	0.294
CK19	120 µm	0.19	0.163	0.002	0.123	0.017	0.826	0.103	0.251
CK19	15 µm	0.095	0.077	0.001	0.074	0.008	0.826	0.057	0.108
CK19	30 µm	0.097	0.086	0.001	0.06	0.011	0.487	0.065	0.136
CK19	60 µm	0.117	0.118	0.001	0.05	0.016	0.356	0.093	0.155
CK19	90 µm	0.144	0.132	0.001	0.087	0.017	0.826	0.098	0.185
EGFR	0 µm	0.079	0.074	0.001	0.063	0.013	0.59	0.036	0.126
EGFR	120 µm	0.073	0.076	0.0	0.021	0.014	0.401	0.06	0.087
EGFR	15 µm	0.088	0.078	0.001	0.089	0.012	0.591	0.046	0.096
EGFR	30 µm	0.073	0.062	0.001	0.039	0.012	0.342	0.051	0.088
EGFR	60 µm	0.087	0.074	0.001	0.057	0.014	0.59	0.065	0.096
EGFR	90 µm	0.085	0.076	0.001	0.054	0.013	0.411	0.063	0.091
ER	0 µm	0.177	0.162	0.001	0.071	0.02	0.654	0.124	0.21
ER	120 µm	0.131	0.128	0.001	0.066	0.017	0.654	0.071	0.174
ER	15 µm	0.109	0.096	0.001	0.071	0.011	0.386	0.065	0.151
ER	30 µm	0.125	0.112	0.001	0.062	0.011	0.722	0.074	0.176
ER	60 µm	0.13	0.113	0.001	0.067	0.011	0.527	0.077	0.194
ER	90 µm	0.148	0.131	0.002	0.098	0.02	0.658	0.099	0.165
Ecad	0 µm	0.146	0.144	0.001	0.075	0.019	0.47	0.107	0.185
Ecad	120 µm	0.149	0.135	0.001	0.08	0.013	0.419	0.128	0.157
Ecad	15 µm	0.128	0.115	0.001	0.076	0.013	0.418	0.093	0.151
Ecad	30 µm	0.134	0.11	0.001	0.083	0.013	0.47	0.101	0.143
Ecad	60 µm	0.157	0.137	0.002	0.1	0.014	0.434	0.129	0.148
Ecad	90 µm	0.143	0.129	0.001	0.089	0.013	0.418	0.117	0.147
HER2	0 µm	0.063	0.054	0.001	0.045	0.01	0.706	0.049	0.075

HER2	120 µm	0.109	0.1	0.001	0.049	0.037	0.296	0.069	0.135
HER2	15 µm	0.088	0.089	0.001	0.039	0.01	0.325	0.062	0.117
HER2	30 µm	0.1	0.086	0.001	0.049	0.01	0.306	0.066	0.118
HER2	60 µm	0.107	0.104	0.001	0.043	0.01	0.517	0.071	0.126
HER2	90 µm	0.108	0.1	0.001	0.049	0.034	0.706	0.07	0.129
Ki67	0 µm	0.08	0.076	0.001	0.043	0.014	0.491	0.058	0.093
Ki67	120 µm	0.1	0.093	0.001	0.05	0.015	0.216	0.065	0.127
Ki67	15 µm	0.075	0.085	0.0	0.03	0.013	0.182	0.055	0.094
Ki67	30 µm	0.086	0.094	0.001	0.04	0.013	0.203	0.054	0.119
Ki67	60 µm	0.101	0.103	0.001	0.048	0.014	0.191	0.065	0.145
Ki67	90 µm	0.108	0.106	0.001	0.049	0.015	0.491	0.076	0.13
PR	0 µm	0.182	0.131	0.002	0.118	0.017	0.455	0.113	0.253
PR	120 µm	0.135	0.128	0.001	0.087	0.014	0.454	0.084	0.157
PR	15 µm	0.129	0.12	0.001	0.075	0.015	0.318	0.084	0.178
PR	30 µm	0.133	0.12	0.001	0.086	0.013	0.322	0.063	0.193
PR	60 µm	0.13	0.111	0.001	0.08	0.006	0.328	0.081	0.175
PR	90 µm	0.138	0.132	0.001	0.093	0.013	0.389	0.084	0.153
Vimentin	0 µm	0.171	0.165	0.001	0.052	0.025	0.505	0.143	0.199
Vimentin	120 µm	0.207	0.18	0.002	0.113	0.025	0.578	0.156	0.24
Vimentin	15 µm	0.126	0.126	0.001	0.041	0.016	0.504	0.109	0.157
Vimentin	30 µm	0.157	0.158	0.001	0.064	0.023	0.413	0.136	0.189
Vimentin	60 µm	0.164	0.166	0.001	0.063	0.023	0.505	0.14	0.205
Vimentin	90 µm	0.187	0.172	0.001	0.097	0.026	0.505	0.147	0.219
aSMA	0 µm	0.105	0.111	0.001	0.038	0.022	0.327	0.095	0.129
aSMA	120 µm	0.107	0.116	0.001	0.041	0.014	0.438	0.093	0.129

aSMA	15 µm	0.098	0.098	0.001	0.062	0.015	0.439	0.079	0.111
aSMA	30 µm	0.097	0.11	0.001	0.035	0.013	0.248	0.084	0.121
aSMA	60 µm	0.104	0.115	0.001	0.037	0.013	0.438	0.098	0.127
aSMA	90 µm	0.13	0.118	0.001	0.084	0.013	0.557	0.112	0.145
p21	0 µm	0.122	0.092	0.002	0.125	0.016	0.487	0.07	0.104
p21	120 µm	0.091	0.095	0.001	0.045	0.014	0.198	0.052	0.122
p21	15 µm	0.081	0.093	0.001	0.038	0.011	0.197	0.052	0.111
p21	30 µm	0.09	0.1	0.001	0.04	0.014	0.487	0.056	0.121
p21	60 µm	0.096	0.109	0.001	0.039	0.013	0.487	0.054	0.129
p21	90 µm	0.104	0.107	0.001	0.07	0.015	0.487	0.054	0.123
pERK	0 µm	0.103	0.112	0.001	0.053	0.017	0.65	0.072	0.129
pERK	120 µm	0.105	0.092	0.001	0.069	0.015	0.65	0.06	0.132
pERK	15 µm	0.101	0.088	0.001	0.096	0.013	0.651	0.063	0.107
pERK	30 µm	0.1	0.098	0.001	0.06	0.014	0.65	0.065	0.133
pERK	60 µm	0.109	0.108	0.001	0.067	0.015	0.389	0.064	0.138
pERK	90 µm	0.114	0.111	0.001	0.067	0.015	0.65	0.064	0.138
pRB	0 µm	0.155	0.102	0.002	0.138	0.015	0.549	0.092	0.144
pRB	120 µm	0.11	0.108	0.001	0.04	0.015	0.336	0.089	0.12
pRB	15 µm	0.095	0.098	0.001	0.033	0.012	0.468	0.073	0.113
pRB	30 µm	0.109	0.098	0.001	0.045	0.011	0.548	0.087	0.146
pRB	60 µm	0.112	0.107	0.001	0.054	0.013	0.286	0.083	0.122
pRB	90 µm	0.112	0.107	0.001	0.052	0.014	0.419	0.1	0.124

Supplementary Table 23: Supplementary Figure 8 Spatial performance of AE

Marker	Model	Mean	Median	SEM	SD	Min	Max	Q1 (25%)	Q3 (75%)
AR	0 µm	0.124	0.101	0.001	0.07	0.015	0.291	0.08	0.17
AR	120 µm	0.097	0.092	0.001	0.048	0.019	0.252	0.057	0.127
AR	15 µm	0.099	0.101	0.001	0.051	0.012	0.269	0.059	0.135
AR	30 µm	0.096	0.076	0.001	0.056	0.018	0.258	0.052	0.12
AR	60 µm	0.106	0.093	0.001	0.056	0.018	0.248	0.064	0.141
AR	90 µm	0.099	0.091	0.001	0.058	0.019	0.241	0.06	0.129
CD45	0 µm	0.099	0.073	0.001	0.055	0.017	0.318	0.06	0.139
CD45	120 µm	0.113	0.082	0.001	0.063	0.044	0.318	0.064	0.16
CD45	15 µm	0.092	0.079	0.001	0.05	0.016	0.234	0.056	0.122
CD45	30 µm	0.095	0.08	0.001	0.048	0.019	0.244	0.059	0.13
CD45	60 µm	0.098	0.078	0.001	0.056	0.019	0.267	0.063	0.117
CD45	90 µm	0.114	0.087	0.001	0.063	0.023	0.318	0.066	0.165
CK14	0 µm	0.132	0.133	0.001	0.055	0.019	0.265	0.09	0.185
CK14	120 µm	0.11	0.071	0.002	0.07	0.02	0.253	0.053	0.162
CK14	15 µm	0.133	0.127	0.001	0.064	0.021	0.281	0.069	0.191
CK14	30 µm	0.113	0.089	0.002	0.075	0.023	0.269	0.046	0.191
CK14	60 µm	0.116	0.104	0.002	0.072	0.024	0.24	0.05	0.17
CK14	90 µm	0.111	0.09	0.002	0.07	0.03	0.273	0.048	0.176
CK17	0 µm	0.084	0.068	0.001	0.039	0.008	0.212	0.063	0.095
CK17	120 µm	0.085	0.068	0.001	0.038	0.008	0.188	0.063	0.1
CK17	15 µm	0.078	0.065	0.001	0.044	0.007	0.307	0.061	0.08
CK17	30 µm	0.083	0.069	0.001	0.042	0.008	0.197	0.062	0.084
CK17	60 µm	0.078	0.068	0.001	0.034	0.007	0.212	0.062	0.077
CK17	90 µm	0.081	0.066	0.001	0.038	0.032	0.307	0.06	0.095

CK19	0 μm	0.208	0.235	0.001	0.077	0.029	0.33	0.141	0.274
CK19	120 μm	0.184	0.204	0.002	0.085	0.024	0.328	0.101	0.262
CK19	15 μm	0.193	0.188	0.002	0.077	0.047	0.319	0.136	0.27
CK19	30 μm	0.189	0.198	0.002	0.075	0.031	0.29	0.131	0.259
CK19	60 μm	0.181	0.199	0.002	0.074	0.046	0.315	0.098	0.24
CK19	90 μm	0.198	0.216	0.002	0.087	0.027	0.313	0.098	0.276
EGFR	0 μm	0.094	0.085	0.001	0.054	0.016	0.342	0.049	0.157
EGFR	120 μm	0.075	0.072	0.001	0.035	0.016	0.342	0.052	0.104
EGFR	15 μm	0.087	0.087	0.001	0.052	0.015	0.206	0.045	0.116
EGFR	30 μm	0.086	0.082	0.001	0.046	0.019	0.21	0.052	0.114
EGFR	60 μm	0.077	0.072	0.001	0.035	0.017	0.239	0.045	0.101
EGFR	90 μm	0.072	0.066	0.001	0.033	0.018	0.292	0.047	0.097
ER	0 μm	0.167	0.157	0.001	0.055	0.019	0.282	0.138	0.211
ER	120 μm	0.155	0.141	0.002	0.078	0.014	0.327	0.117	0.194
ER	15 μm	0.141	0.147	0.001	0.055	0.016	0.328	0.13	0.152
ER	30 μm	0.136	0.157	0.001	0.064	0.015	0.328	0.103	0.188
ER	60 μm	0.144	0.148	0.002	0.074	0.016	0.316	0.102	0.185
ER	90 μm	0.149	0.155	0.001	0.047	0.016	0.282	0.118	0.186
Ecad	0 μm	0.143	0.147	0.001	0.064	0.017	0.313	0.121	0.16
Ecad	120 μm	0.152	0.153	0.002	0.072	0.016	0.37	0.143	0.178
Ecad	15 μm	0.136	0.136	0.001	0.056	0.017	0.246	0.123	0.152
Ecad	30 μm	0.143	0.132	0.002	0.073	0.016	0.332	0.121	0.151
Ecad	60 μm	0.149	0.147	0.002	0.067	0.017	0.346	0.132	0.167
Ecad	90 μm	0.14	0.148	0.002	0.069	0.015	0.351	0.125	0.152
HER2	0 μm	0.069	0.058	0.001	0.041	0.009	0.36	0.046	0.075

HER2	120 µm	0.099	0.089	0.001	0.048	0.036	0.325	0.069	0.127
HER2	15 µm	0.081	0.074	0.001	0.035	0.009	0.188	0.065	0.112
HER2	30 µm	0.084	0.077	0.001	0.035	0.01	0.197	0.063	0.115
HER2	60 µm	0.088	0.08	0.001	0.041	0.009	0.306	0.063	0.118
HER2	90 µm	0.101	0.098	0.001	0.046	0.038	0.232	0.067	0.12
Ki67	0 µm	0.074	0.082	0.0	0.031	0.014	0.281	0.056	0.097
Ki67	120 µm	0.088	0.089	0.001	0.042	0.014	0.208	0.061	0.126
Ki67	15 µm	0.075	0.08	0.001	0.029	0.013	0.153	0.061	0.089
Ki67	30 µm	0.085	0.097	0.001	0.035	0.013	0.281	0.059	0.11
Ki67	60 µm	0.107	0.111	0.001	0.059	0.014	0.282	0.07	0.133
Ki67	90 µm	0.097	0.108	0.001	0.039	0.014	0.173	0.07	0.124
PR	0 µm	0.133	0.13	0.001	0.06	0.017	0.345	0.089	0.193
PR	120 µm	0.09	0.089	0.001	0.044	0.017	0.318	0.058	0.128
PR	15 µm	0.12	0.116	0.002	0.066	0.016	0.307	0.078	0.168
PR	30 µm	0.106	0.099	0.001	0.042	0.017	0.318	0.083	0.136
PR	60 µm	0.113	0.115	0.001	0.049	0.018	0.318	0.085	0.152
PR	90 µm	0.111	0.099	0.001	0.056	0.018	0.303	0.073	0.149
Vimentin	0 µm	0.14	0.137	0.001	0.051	0.039	0.33	0.111	0.181
Vimentin	120 µm	0.195	0.217	0.002	0.066	0.056	0.324	0.187	0.242
Vimentin	15 µm	0.165	0.177	0.001	0.044	0.037	0.291	0.141	0.197
Vimentin	30 µm	0.159	0.171	0.001	0.045	0.053	0.284	0.127	0.186
Vimentin	60 µm	0.174	0.193	0.001	0.058	0.056	0.294	0.154	0.209
Vimentin	90 µm	0.17	0.187	0.001	0.058	0.056	0.321	0.128	0.214
aSMA	0 µm	0.131	0.129	0.001	0.052	0.029	0.329	0.116	0.15
aSMA	120 µm	0.161	0.149	0.002	0.076	0.037	0.331	0.12	0.202

aSMA	15 µm	0.132	0.139	0.001	0.035	0.027	0.228	0.108	0.158
aSMA	30 µm	0.112	0.124	0.001	0.036	0.03	0.222	0.09	0.14
aSMA	60 µm	0.128	0.135	0.001	0.043	0.032	0.273	0.111	0.16
aSMA	90 µm	0.128	0.13	0.001	0.05	0.032	0.279	0.108	0.157
p21	0 µm	0.072	0.069	0.0	0.028	0.016	0.247	0.054	0.097
p21	120 µm	0.08	0.082	0.001	0.035	0.019	0.198	0.055	0.106
p21	15 µm	0.079	0.087	0.001	0.033	0.017	0.15	0.056	0.104
p21	30 µm	0.086	0.097	0.001	0.038	0.019	0.159	0.055	0.113
p21	60 µm	0.09	0.104	0.001	0.036	0.016	0.186	0.056	0.124
p21	90 µm	0.087	0.096	0.001	0.037	0.02	0.183	0.061	0.117
pERK	0 µm	0.101	0.109	0.001	0.048	0.017	0.344	0.067	0.141
pERK	120 µm	0.094	0.076	0.001	0.052	0.017	0.321	0.062	0.13
pERK	15 µm	0.087	0.071	0.001	0.041	0.016	0.214	0.064	0.119
pERK	30 µm	0.101	0.095	0.001	0.049	0.018	0.257	0.071	0.125
pERK	60 µm	0.101	0.098	0.001	0.053	0.017	0.228	0.062	0.126
pERK	90 µm	0.101	0.086	0.001	0.051	0.018	0.257	0.063	0.132
pRB	0 µm	0.111	0.115	0.001	0.054	0.016	0.283	0.069	0.157
pRB	120 µm	0.086	0.081	0.001	0.036	0.015	0.204	0.067	0.107
pRB	15 µm	0.101	0.093	0.001	0.035	0.013	0.196	0.073	0.14
pRB	30 µm	0.105	0.102	0.001	0.029	0.018	0.165	0.085	0.126
pRB	60 µm	0.093	0.082	0.001	0.04	0.016	0.172	0.07	0.118
pRB	90 µm	0.092	0.091	0.001	0.034	0.016	0.199	0.068	0.115

SSupplementary Table 24: Supplementary Figure 9 Spatial performance of AE M
